# Impact of Yogurt and Rolled Oats Consumption on the Gut Microbiome: A Randomized Crossover Study Displaying Individual Responses and General Resilience

**DOI:** 10.1016/j.tjnut.2026.101408

**Published:** 2026-02-11

**Authors:** Kerstin Thriene, Virginie Stanislas, Kun D Huang, Till Strowig, Karin B Michels

**Affiliations:** 1 Institute for Prevention and Cancer Epidemiology, Faculty of Medicine and Medical Center, University of Freiburg, Freiburg, Germany; 2 Institute of Microbiology, Friedrich Schiller University, Jena, Germany; 3 Department of Microbial Immune Regulation, Helmholtz Center for Infection Research, Braunschweig, Germany; 4 Centre for Individualised Infection Medicine (CiiM), a joint venture between the Helmholtz-Centre for Infection Research (HZI) and the Hannover Medical School (MHH), Hannover, Germany; 5 Cluster of Excellence Balance of the Microverse, Friedrich Schiller University, Jena, Germany

**Keywords:** yogurt, rolled oats, gut microbiome, randomized crossover intervention study, probiotic, prebiotic, fermented food

## Abstract

**Background:**

Yogurt and rolled oats are commonly linked to gut health through probiotic and prebiotic effects, but these potential benefits remain insufficiently studied, especially in healthy individuals.

**Objectives:**

This study primarily aimed to investigate the effects of daily yogurt and rolled oats consumption on gut microbial composition. Secondary outcomes included stool metabolites and blood-based health markers.

**Methods:**

In this randomized, open-label, 2-period crossover trial, 119 healthy participants were randomly assigned to 1 of 2 sequences: 250 g of yogurt daily followed by 250 g of yogurt with 50 g of rolled oats, or the reverse with a washout period in between. Stool and blood samples were collected at baseline and post intervention. Metagenomic sequencing and metabolomic analyses were conducted on stool samples, whereas health markers related to metabolic control, inflammation, immune response, oxidative stress, and gut permeability were assessed in the participants’ blood.

**Results:**

Of the 119 randomly divided participants, 110 completed the study (53 yogurt first, 57 yogurt and rolled oat first). Yogurt consumption transiently increased yogurt-associated bacteria, with *Streptococcus thermophilus* rising from absent to 0.97% [95% confidence interval (CI): 0.71, 1.26] in the yogurt intervention and 0.79% (95% CI: 0.58, 1.03) in the yogurt with oats intervention. In a small *Prevotella*-predominant subgroup (*n* = 8), adding rolled oats increased microbial evenness (*q* < 0.001) and reduced interindividual divergence (*q* < 0.05), suggesting a temporary slight homogenization. No additional effects on fecal short-chain fatty acids concentrations or human health markers were identified. Functional metagenomic changes were mainly driven by yogurt-derived bacterial enrichment.

**Conclusions:**

A healthy gut microbiota is largely stable and resilient to short-term diet changes, yet individual differences highlight the importance of personalized dietary recommendations.

**Clinical Trial Registration No. (German Trial Register):** DRKS00023146 (https://drks.de/search/en/trial/DRKS00023146/details).

## Introduction

The human gut microbiome is a complex ecosystem composed of trillions of microbes, including bacteria, fungi, and archaea, residing primarily in the nutrient-rich gastrointestinal tract. Despite its unique composition in each individual, a few main phyla—Firmicutes (Bacillota), Bacteroidetes (Bacteroidota), Actinobacteria (Actinomycetota), Proteobacteria (Pseudomonadota), Fusobacteria (Fusobacteriota), and Verrucomicrobia (Verrucomicrobiota)—constitute the core microbiome [1]. This core microbiome is established in early childhood and remains stable over long periods, yet it is highly malleable, continuously remodeling in response to environmental changes [[2], [3], [4]]. Nutrition significantly influences the microbiome for example by modulating the relative abundance of individual species within an established microbial community, or by introducing new species into the gut microbiome. Dietary interventions with probiotic and prebiotic foods have been shown to alter microbiota composition, potentially improving health outcomes [5]. Probiotics and prebiotics, often termed functional foods, have garnered significant public interest in recent years [6,7]. These products, which include supplements and fermented foods, are marketed for their health benefits, although not all fermented foods meet the criteria for probiotics. In fact, some intervention studies have shown only minor effects of consuming fermented foods, such as sauerkraut, on gut microbiome composition [8]. To clearly distinguish probiotic foods, the term “probiotics” refers to live microorganisms, such as those found in yogurt, which provide health benefits, including improved lactose digestion and enhanced immune function [9].

Including probiotic cultures in yogurt can enhance its functional properties, potentially reducing the risk of nutrition-related diseases [10]. For instance, probiotic yogurt consumption has been associated with improved lactose tolerance, immune enhancement, and prevention of gastrointestinal disorders [11]. To date, there is still limited research on the impact of yogurt consumption on gut microbiome composition in humans. One study involving 10 children infected with *Helicobacter pylori* demonstrated that yogurt consumption improved microbiome composition and reduced *H. pylori* load, suggesting a potential use of yogurt in disease management [12]. However, large and robust human intervention studies are lacking in investigating the potential effects of yogurt as a probiotic food on healthy gut microbiomes.

Prebiotics, such as those found in rolled oats, provide a nutritional source for gut microorganisms [13]. These complex carbohydrates, including fibers that comprise 10% of oats, are not metabolized by human digestive enzymes and thus reach the colon where microbial enzymes break them down [14,15]. The host can take up the resulting metabolites, such as short-chain fatty acids (SCFAs), promoting various health benefits [13,16]. Oat fibers, particularly β-glucan, have been shown to lower LDL cholesterol and improve cardiovascular health, although the exact mechanisms remain unclear [[17], [18], [19]]. Rolled oats are a popular breakfast staple known for their benefits, including reducing the risk of cardiovascular disease. However, oat fibers’ specific role and interaction with gut microorganisms warrant further investigation.

To evaluate the effects of yogurt and rolled oats consumption on the human gut microbiome and overall human health, we conducted a randomized crossover study with 110 healthy participants, including washout phases between interventions. This design minimizes individual variability through within-subject comparisons, reduces confounding factors, and enhances statistical power, ensuring more reliable and robust results. Participants consumed 250 g of yogurt daily for 4 wk during one intervention and 250 g of yogurt combined with 50 g of rolled oats during a second 4-wk intervention. We examined changes in the gut microbiome, metabolic activity, and blood-based health markers in response to the dietary interventions. In particular, we analyzed inflammation markers such as high-sensitivity C-reactive protein (hsCRP), IL-6, and tumor necrosis factor receptor 2 (TNFR2), fructosamine as a marker for effects on blood sugar, markers for oxidative stress such as receptor for advanced glycation end products (RAGE) and 8-hydroxy-2′-deoxyguanosine (8-OHdG) as well as zonulin as a marker for gut permeability. Understanding these interactions could provide valuable insights into the health benefits of these common dietary components and their role in promoting a healthy gut microbiome.

## Methods

### Study design

**FIGURE 1:**
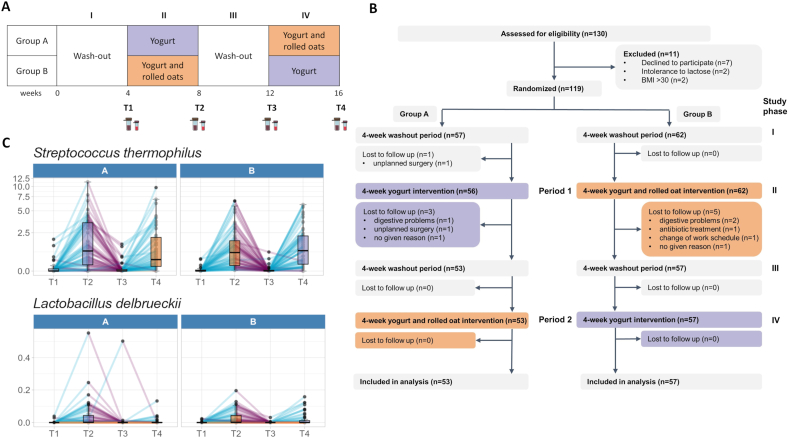
Temporary increase in yogurt bacteria abundance in the human gut microbiota after yogurt consumption. (A) Study design. Participants consumed either 250 g/d of yogurt or 250 g/d of yogurt and 50 g/d of rolled oats during the intervention periods. Stool and blood samples were collected at the end of each study phase. (B) Flowchart of study participant enrolment. (C) Relative abundance of yogurt bacteria (*Streptococcus thermophilus* and *Lactobacillus delbrueckii*) in stool samples for each study phase.

A 16-wk randomized dietary intervention study with a crossover design was conducted, consisting of a 4-wk washout phase (baseline), followed by a first 4-wk intervention, a second 4-wk washout phase (baseline), and a second 4-wk intervention (Figure 1A). During the washout phases, no yogurt or rolled oats were consumed. The washout phases served to eliminate the effects of previously consumed yogurt and rolled oats and to minimize carryover effects between intervention phases. There is currently no consensus on the optimal duration of washout phases in dietary intervention studies [20]. Reported durations in crossover designs vary widely, typically between 2 and 6 wk [21], and some studies have reported that microbiota composition can return to baseline in a matter of days [22]. In our study, a 4-wk washout was chosen as a compromise between available evidence and practicability, supporting participant compliance while minimizing carryover effects. A total of 119 healthy volunteers were recruited in Freiburg im Breisgau, Germany. Microbiome studies aiming to detect differences in microbiome diversity or changes in microbial composition do not allow precise power calculations because of the complexity of the data [23,24]. Therefore, the sample size was chosen based on comparable human intervention studies that reported detectable effects of yogurt on gut microbiome composition: a parallel study with 72 *H. pylori*–infected children and a crossover study with 63 adults [25,26]. On the basis of these previous findings and applying high-resolution sequencing methods in our analysis, we aimed to include 100 healthy participants in our study, assuming a 10% dropout rate (total target: 120 participants, 60 in each intervention group). Recruiting and study started in 2020 and lasted until 2021 when the last participants finished their last intervention.

The random allocation sequence for the crossover sequences was generated and implemented in a blinded manner using a secure, computerized randomization program (clinical trial randomization tool, National Cancer Institute Division of Cancer Prevention: https://ctrandomization.cancer.gov/). Allocation was concealed from investigators responsible for enrolment and was performed digitally only after participants were deemed eligible and enrolled, before the first intervention period. In group A, participants consumed 250 g/d of yogurt (3.8% fat, Schwarzwaldmilch GmbH) during the first intervention period and 250 g/d of yogurt and 50 g/d of rolled oats (Peter Kölln GmbH & Co. KGaA) during the second intervention period, whereas in group B, the interventions were carried out in reverse order. The study was conducted as an open-label trial, as both participants and investigators were aware of the interventions throughout the study. Participants were instructed to avoid additional yogurt and rolled oats products, prebiotic and probiotic supplements, and fermented foods throughout the study. Both yogurt and rolled oats were purchased commercially for standardization purposes. Once in each of the 4 study phases, participants completed a 24-h dietary recall to document their intake and to verify adherence to the study diet, including avoidance of additional probiotic or oat products. On the basis of these assessments, no participants were excluded from the analysis. Online questionnaires were provided by the Human Study Site of the German Institute for Nutritional Research Potsdam-Rehbrücke (Deutsche Institut für Ernährungsforschung), Potsdam, Germany. At the end of each study phase, stool and blood samples were collected from each participant. Potential adverse events were defined before study initiation. Participants were monitored at each visit for gastrointestinal symptoms, infections, and other health complaints. However, no harm was anticipated, as the dietary components used in the intervention (yogurt and rolled oats) are commonly consumed foods that are readily available in supermarkets. Exclusion criteria included a history of chronic diseases, acute or chronic gastrointestinal symptoms (e.g., inflammatory bowel disease), 1 or more episodes of strong diarrhea, intake of oral probiotic or prebiotic supplements or any antibiotics within the past 3 mo before the begin of the study, smoking, consumption of >2 standard drinks of alcohol (20 g pure alcohol in total) per day, a BMI <19 kg/m^2^ or >30 kg/m^2^, major dietary restrictions such as veganism, raw-food-only consumption or food allergies and intolerances (e.g., lactose intolerance), or planned changes of dietary habits within the study timeframe. No changes were made to the eligibility criteria or any other aspects of the trial design after the study commenced.

The primary outcome of the study was the effect of yogurt and rolled oats consumption on the gut microbiome in healthy participants assessed by metagenomic analysis of stool samples before and after each intervention. This outcome was chosen as primary because it directly reflects the main target of the interventions. Secondary outcomes included the effects of yogurt and rolled oats consumption on microbial metabolites in stool and inflammatory markers in blood, providing additional mechanistic information. Changes in gut microbiome composition are expected to occur first and may then lead to changes in metabolites and blood markers. No changes in outcomes were made after the start of the study.

All participants provided written informed consent. This study was approved by the ethics committee of the Albert-Ludwigs-University Freiburg (application number 20-1035; Trial protocol available on request). Patients and/or the public were not involved in the design, or conduct, or reporting, or dissemination plans of this research.

### Biospecimen collection and analyses

Blood samples were obtained at the study center (Institute for Prevention and Cancer Epidemiology, Faculty of Medicine and Medical Center of the University of Freiburg, Germany) at the end of each of the 4 study phases. Fructosamine and hsCRP were analyzed at the Institute for Clinical Chemistry and Laboratory Medicine, Medical Centre, University of Freiburg, Germany. IL-6, TNFR2, RAGE, 8-OHdG, and zonulin were analyzed at the Boston Children’s Hospital, Boston, United States. IL-6, TNFR2, RAGE, and 8-OHdG were analyzed from plasma by an ELISA assay (R & D Systems), zonulin was analyzed from serum by a competitive enzyme immunoassay (ALPCO Diagnostics).

Stool samples were collected by participants at home in 2 containers (*1*) native, *2*) stabilized with 96% ethanol). Participants were instructed to store the samples in the refrigerator after collection and to return them to the study center within 24 h. At the study center, samples were immediately aliquoted and stored at −80°C until subsequent analysis. For analysis of SCFA, native stool samples were processed by Metabolon Inc, using ultra-HPLC/MS/MS. Stabilized stool samples were processed for metagenome analysis. DNA was extracted using the ZymoBIOMICS 96 MagBead DNA Kit (D4308). Libraries were constructed using Illumina DNA PCR-Free Prep Kit without size selection. Libraries were sequenced on the Illumina NovaSeq 6000 S4 system with a target depth of 25,000,000 reads/sample.

### Preprocessing, taxonomic, and functional profiling of metagenomic samples

All metagenomic samples in this study were first subjected to raw sequence preprocessing using BBMap quality control pipeline (https://sourceforge.net/projects/bbmap/), which removed read bases with quality score <10 and reads from human host (the Ensembl masked human genome GRCh38) and phiX contamination (*minid* = 0.95 *maxindel* = 3 *bwr* = 0.16 *bw* = 12 *quickmatch fast minhits* = 2 *qtrim = rl trimq* = 10). Afterward, for each sample, the species-level microbial community was profiled and the abundance of each microbial member was quantified, using MetaPhlAn 4 [27] with *-t rel_ab_w_read_stats –force –sgb*. Similarly, HUMAnN 3 [28] was performed with default settings to quantify microbial metabolic pathways and gene contents in each sample. Next, we also renormalized the abundance quantifications to copies per million (CPM) unit using the utility script *humann_renorm_table.py* with *-u cpm*, followed by converting individual functional profiles into a merged abundance table using the utility script *humann_join_table.py*. The merged abundance table was cleaned by retaining only gene contents that were detected in >10% of all samples (946,461 genes) and later on at a minimum 1 CPM in >10% of all samples (191,601 genes). Moreover, to further focus on gene contents previously characterized with Kyoto Encyclopedia of Genes and Genomes (KEGG) [29] and Gene Ontology (GO) [30] databases, we regrouped the default gene contents (191,601 genes) into 967 KEGGs and 2402 GO pathways using the utility script *humann_regroup_table.py* and generated the merged abundance table as described above.

### Statistical analyses

Analyses were performed in R (R Core Team, version 4.3.1). If not specified otherwise, all participants were included in the analysis. For the microbiome analysis, the first 2 samples from 1 participant (T1 and T2) were not correctly prepared and were therefore excluded. Missing values were managed using outcome-specific complete-case analysis. For each outcome, only observations with missing values for that specific variable were excluded from the analysis. The number of missing observations were: TNFR2 (2), IL-6 (2), RAGE (2), 8-OHdG (2), zonulin (3), fructosamine (4), and CRP (5). For SCFAs, all metabolites had 1 missing observation except hexanoic acid and isovaleric acid, which each had 2. For sequencing-based outcomes, values were recorded for all samples. Zero or undetected entries reflect true absence or below-detection-limit measurements rather than missing data, and all available observations were included in the analyses. Uniform Manifold Approximation and Projection (UMAP) was computed on species relative abundances using Euclidean distances. Alpha diversity of each sample was computed on the relative abundances of all species using 6 different measures, observed richness, Pielou and Shannon indices, the Berger-Parker dominance index (dbp), divergence to median, and log modulo skewness. Permutational multivariate analysis of variance (PERMANOVA) analysis was computed based on the Bray-Curtis distances of the relative abundances of the microbial species (adonis2 function, vegan package). The PERMANOVA model was run on the microbiome at baseline (T1) to investigate the contribution of age, gender, BMI, richness, intervention group, and abundance of enterotype genus to the observed beta diversity of the baseline microbiota profiles. Three participants with missing BMI values were excluded of this analysis. In addition, to account for the compositional nature of microbiome data, we also performed PERMANOVA using Aitchison distance (Euclidean distance on centered log-ratio transformed abundances); results were broadly consistent with those obtained using Bray-Curtis distance (data not shown). Analysis of the effect of the intervention was done using mixed models. Linear mixed models were used for alpha diversity measures, blood markers, and metabolites after box-cox transformation. Outliers were removed for the blood marker analysis (1 different individual data point for the analysis on zonulin, CRP, and IL-6, and 4 data points from 1 participant with high values for the analysis on fructosamine). Zero-inflated gaussian mixed model [Negative Binomial and Zero-Inflated Mixed Models (NBZIMM) package [31]] were used after arcsine square root transformation for the analysis of individual species, genes, as well as pathways, KEGGs, and GO terms with presence of zeroes. Analyses were run for species with a prevalence ≥0.03 (species present in ≥3% of samples) and for genes present in ≥10% of samples. For pathways, KEGGs, and GO terms without zeroes, linear mixed models were used after box-cox transformation.

Two types of mixed models were used depending on the comparison:

1) To compare the interventions:

$$Y_{\left\{ijk\right\}}=\mu +\beta_{1}B_{\left\{ijk\right\}}+\beta_{2}T_{\left\{ijk\right\}}+\beta_{3}C1_{\left\{ijk\right\}}+\beta_{4}C2_{\left\{ijk\right\}}+s_{\left\{ik\right\}}+\epsilon_{\left\{ijk\right\}}$$

2) To assess the effect of each intervention separately:

$$Y_{\left\{ijk\right\}}=\mu +\beta_{1}B_{\left\{ijk\right\}}+\beta_{2}P_{\left\{ijk\right\}}+s_{\left\{ik\right\}}+\epsilon_{\left\{ijk\right\}}$$

For i∈{A,B} (sequence), j∈{1,2}(Period 1, Period 2) and $k=1,\ldots ,n_{i}$ (subjects within sequence i), with $n_{A}+n_{B}=110$.

With:

• $Y_{\left\{ijk\right\}}$: response observed for subject k in period j of sequence i.
• $B_{\left\{ijk\right\}}$: 0 for baseline or 1 for other.
• $P_{\left\{ijk\right\}}$: 0 for 1st period and 1 for 2nd period (1st period = Phase II, 2nd period = Phase IV).
• $T_{\left\{ijk\right\}}$: 0 or 1 if after yogurt-rolled oats.
• $C1_{\left\{ijk\right\}}$: indicator for period 2, conditional on receiving yogurt in period 1.
• $C2_{\left\{ijk\right\}}$: indicator for period 2, conditional on receiving yogurt with rolled oats in period 1.
• $s_{\left\{ik\right\}}$: random subject-level effects.

The coefficient of interest for model 1 is $\beta_{2}$: (yogurt and rolled oats compared with baseline) compared with (yogurt compared with baseline), for model 2 is $\beta_{1}$: after intervention compared with baseline.

A more detailed description of the statistical model is provided in the supplement.

We estimated marginal means for each level of the dependent variable of interest (either T or B), generated contrasts, and conducted pairwise comparisons using Tukey tests [32]. *P* values were corrected for multiple hypothesis testing over all simultaneously investigated outcomes (either alpha diversity, blood markers, metabolites or microbiome measures) using Benjamini-Hochberg correction. Model fit for alpha diversity, blood markers, and metabolites was evaluated using tests for overdispersion and 0 inflation, along with residual diagnostics from the DHARMa package [33], with no relevant deviations detected. For microbiome features, NBZIMM with arcsine square root transformation was selected from several candidate models, and model assumptions were assessed through visual inspection of residual distributions for a subset of models. No formal carryover effect was estimated or tested, as formal testing of carryover effects is generally not recommended in 2-period crossover designs because of their close relationship with period effects [34].

Clustering was performed independently for both baseline timepoints (T1 and T3) based on the relative abundance of microbial species with a prevalence of ≥0.03. Two different clustering approaches were used, kmeans clustering on the Bray-Curtis distance matrix and Louvain community detection algorithms on the shared nearest-neighbor graph (NNgraph). Choice of the optimal number of clusters was defined using the silhouette width criteria. Participants who were not attributed to the same cluster between T1 and T3 were defined as misclassified and were removed for subsequent analysis. Mixed models, as previously described, were run separately within each cluster to assess intervention effects in these subgroups. Additionally, an analysis was conducted in which the 2 interventions yogurt and yogurt-rolled oats were merged into 1. Analyses were run for species with a prevalence ≥0.03.

## Results

### Consumption of yogurt leads to a temporary increase in yogurt bacteria abundance in the human gut microbiota

Of the 119 healthy volunteers who enrolled in the study, 110 completed all 4 study phases. Nine participants withdrew because of digestive problems caused by yogurt consumption (*n* = 3), unplanned surgery (*n* = 2), antibiotic treatment (*n* = 1), a change of work schedule (*n* = 1), or for unspecified reasons (*n* = 2) (Figure 1B). The final study population comprised 53 participants (48%) in Group A and 57 participants (52%) in Group B. Characteristics of the participants at the start of the study are depicted in Table 1.

**TABLE 1:** Demographic characteristics in each group at baseline

	A, *n* = 53^1^^,^^2^	B, *n* = 57^1^^,^^2^
Gender		
Male	26 (49%)	25 (44%)
Female	27 (51%)	32 (56%)
Age	40 (27, 52)	40 (28, 55)
BMI	23.00 (21.13, 24.90)	23.82 (21.32, 25.28)
Missing	2	1

1 A, first intervention yogurt; B, first intervention yogurt and rolled oats.

2 *n* (%); median (IQR).

**FIGURE 2:**
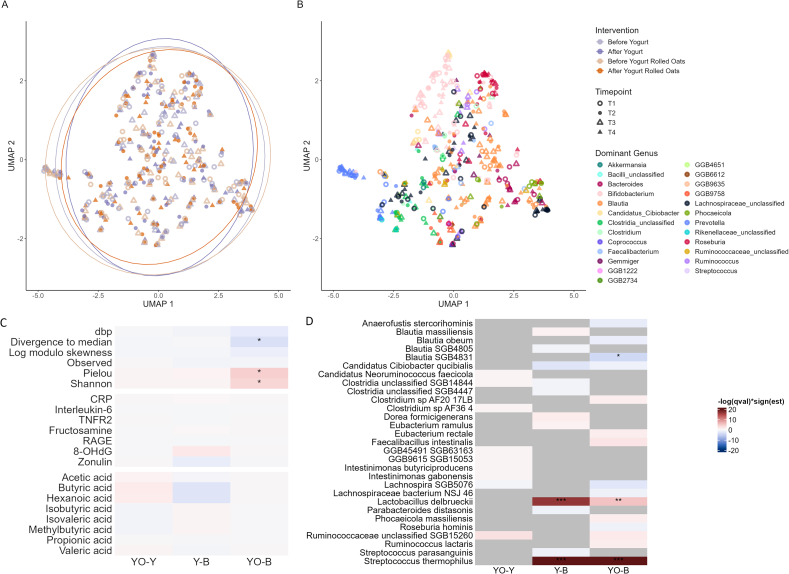
Gut microbial composition, SCFA concentrations in stool, and human health markers after yogurt or yogurt and rolled oats consumption. (A, B) UMAP representation of the microbial composition colored by intervention with 95% data ellipse for each subgroup. Red ellipses represent the yogurt-rolled oats intervention, whereas blue ellipses describe the yogurt intervention (A) or colored by the most dominant genus for each sample (B). (C, D) Effect of each intervention on alpha diversity measures, blood markers, and metabolites concentrations (C) as well as on the relative abundance of each species (species with a *P* value < 0.015 are shown for visualization purposes) (D). The color code (red indicating an increase, blue indicating a decrease) reflects the significance level of the estimated differences between yogurt vs. baseline (Y-B), yogurt-rolled oats compared with baseline (YO-B), yogurt-rolled oats vs. yogurt (YO-Y) based on *q* values adjusted for multiple testing. Statistical significance is indicated as: ∗*q* value < 0.05, ∗∗*q* value < 0.01, ∗∗∗*q* value < 0.001. SCFA, short-chain fatty acid; UMAP, Uniform Manifold Approximation and Projection.

Shotgun metagenomic sequencing generated a mean of 23,426,238 paired reads per sample, taxonomic and functional profiling identified a mean of 212 microbial species and 339,955 gene families per sample. Taxonomic analysis of stool samples revealed a significant enrichment of yogurt-derived bacteria (*Streptococcus thermophilus* and *Lactobacillus delbrueckii*) after the 2 intervention phases in which participants consumed 250 g of yogurt daily, compared with washout phases (Figure 1C and Figure 2D, Supplementary Figure 1). Specifically, *Streptococcus thermophilus* relative abundance increased from baseline (absent) to 0.97 [95% confidence interval (CI): 0.71, 1.26, *q* value < 0.0001] for the yogurt intervention and to 0.79 (95% CI: 0.58, 1.03, *q* value < 0.0001) for the yogurt and rolled oats intervention. *Lactobacillus delbrueckii* showed smaller increases, reaching 0.008 (95% CI: 0.004, 0.013, *q* value < 0.0001) for the yogurt intervention and 0.003 (95% CI: 0.001, 0.006, *q* value = 0.009) for the yogurt and rolled oats intervention. All values were back-transformed from arcsine square root–transformed model coefficients, assuming taxa were absent at baseline. In our study, the additional consumption of 50 g of rolled oats daily together with yogurt exhibited no significant effect on the abundance of the 2 yogurt bacteria in the stool samples (Figure 2D, Supplementary Figure 1).

Our results indicate that consuming 250 g of yogurt daily for 4 wk temporarily enriches the yogurt bacteria in the gut microbiota, whereas an additional 50 g of rolled oats daily does not seem to affect this result.

### No major effects of yogurt or yogurt and rolled oats consumption on gut microbial composition, SCFA concentrations in stool, and human health markers

Next, we aimed to determine whether the enrichment of yogurt bacteria in the gut microbiome, after yogurt consumption, affects other aspects of the host. Consequently, we examined the impact of yogurt, or yogurt combined with rolled oats, on gut microbial composition, fecal SCFA concentrations, and human health-associated markers.

Differences in bacterial composition between samples at baseline are mostly determined by the individual microbial signatures of the participants. Specifically, the relative abundance of single bacterial genera known to characterize different human gut microbiome profiles (*Prevotella*, *Bacteroides*, and *Ruminococcus*) and the observed richness are significantly associated with differences in bacterial community composition at baseline (Supplementary Table 1). In contrast, individual host characteristics such as BMI, age, gender, or interventional group are not significantly associated. However, each of the significant variables explains <5% of the total variance, with 78% of the total variance remaining unexplained. To confirm the robustness of these results, we also performed PERMANOVA using Aitchison distance. The findings were broadly consistent with those obtained using Bray-Curtis distance, with observed, Prevotella, and Bacteroides remaining significant, whereas BMI showed a modest association (data not shown). UMAP representations of the microbial composition also illustrate these results. The microbial samples are grouped according to the predominant genus, whereas no clear grouping according to population characteristics can be observed (Figure 2B, Supplementary Figure 2 and 3).

UMAP representation of the microbial composition does not reveal substantial microbial community changes comparing stool samples before and after each intervention (Figure 2A). Most participants displayed a stable microbiome throughout the study, with only a few showing major shifts (Supplementary Figure 2F). To more precisely examine compositional changes in the gut microbiota resulting from both interventions, we investigated changes in the relative abundance of individual species (Figure 2D, Supplementary Figure 1 and Supplementary Figure 4) and 6 different alpha diversity metrics (Figure 2C, Supplementary Figure 5). After the 4-wk intervention with yogurt and rolled oats, a slight increase in the evenness indices (Shannon and Pielou) (*q* value < 0.05) and a slight decrease in divergence to the median (*q* value < 0.05) were detected compared with the value at baseline. These shifts indicate that species distribution within each sample tends to become more uniform. At the same time, the samples become increasingly similar, suggesting a homogenization of the gut microbial composition between participants after 4 wk of yogurt and rolled oats consumption. However, these shifts were not observed after the 4-wk yogurt intervention without rolled oats. No significant shifts in the number of observed species (observed richness) or relative abundance of the sample’s most and least abundant species (dbp and log modulo skewness) were detected after either intervention.

In general, very few changes in the gut microbial composition at the species level were observed after consuming yogurt or yogurt in combination with rolled oats (Figure 2D, Supplementary Figures 1 and 4). In fact, only the 2 bacterial species present in yogurt, *Streptococcus thermophilus* and *Lactobacillus delbrueckii*, were significantly enriched directly after each of the 2 interventions. The only other significant change was observed for *Blautia SGB4831*, for which the relative abundance significantly decreased after the yogurt-rolled oats intervention but not after the yogurt-only intervention.

Additionally, we analyzed the concentrations of microbial-derived SCFAs in the stool samples at each of the 4 time points, but none of the metabolites analyzed displayed significant changes (Figure 2C, Supplementary Figure 6). Furthermore, we were interested in the effect of yogurt and rolled oats consumption on markers of human health. Therefore, we analyzed inflammation markers such as CRP, IL-6, and TFNR2, fructosamine as a marker for effects on blood sugar, markers for oxidative stress such as RAGE and 8-OHdG as well as zonulin as a marker for gut permeability. Also, none of these markers showed significant changes after consuming yogurt or yogurt and rolled oats (Figure 2C, Supplementary Figure 7).

In summary, the results of our study suggest that the 4-wk consumption of yogurt or yogurt in combination with rolled oats does not cause major changes in the gut microbiome composition in healthy individuals (apart from the enrichment of yogurt-derived bacteria). Only a slight homogenization of the composition of the gut microbiome intra and between participants was observed after the yogurt and rolled oats intervention. No significant changes were observed in the SCFA produced by the gut microbiota or in human health markers either.

### Cluster-specific effects of yogurt and rolled oats consumption on gut microbial composition and short-chain fatty acid concentrations

**FIGURE 3:**
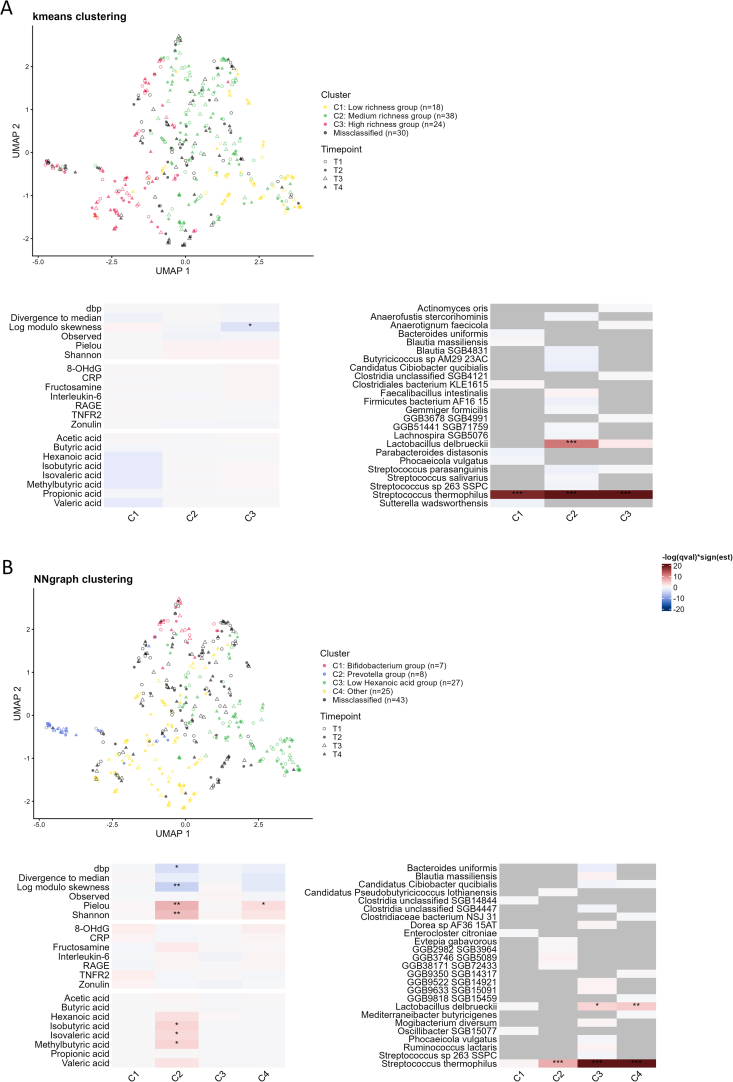
Effect of both interventions within groups of participants sharing similar baseline microbiome. Groups are either defined by (A) kmeans clustering or (B) NNgraph clustering. For each clustering, a UMAP plot depicts the organization of the clusters in the overall gut microbiota species composition space. Two heatmaps represent the merged effect of the 2 interventions (after vs. before) on the alpha diversity measures, blood markers, metabolites concentrations, and relative abundance of each species. Species with a *P* value < 0.015 are shown for visualization purposes. The color code (red increase, blue decrease) reflects the significance level of the estimated differences between after vs. before in each cluster based on *q* values adjusted for multiple testing. Statistical significance is indicated as: ∗*q* value < 0.05, ∗∗*q* value < 0.01, ∗∗∗*q* value < 0.001.

Next, we investigated whether different types of responders exist within the study population. Because the gut microbial signature is the primary factor driving differences between samples at baseline, we defined subpopulations based on their gut microbial profile at baseline using 2 different clustering approaches (kmeans and NNgraph). We investigated the response to both interventions in each identified cluster. Kmeans clustering (Figure 3A) revealed 3 clusters mainly characterized by different levels of microbial richness. Cluster 1 (18 participants) includes samples displaying low observed richness, low Shannon diversity, and low concentrations of hexanoic acid (Supplementary Figure 8). In cluster 2 (38 participants), samples with medium levels of observed richness and low divergence to median are grouped together, whereas in cluster 3 (30 participants), samples with high levels of observed richness are combined (Supplementary Figure 8). A total of 30 participants were defined as misclassified and were not attributed to any subpopulation. A significant decrease in the concentrations of isobutyric acid, isovaleric acid, methylbutyric acid was observed in cluster 1 after the yogurt-rolled oats intervention (*q* value < 0.05) (Supplementary Figure 9). In clusters 2 and 3, no additional significant changes were detected after either of the 2 interventions. Additionally, a substantial decrease in the concentration of rare species (log modulo skewness) could be detected in cluster 3 (*q* value < 0.05) when the effects of the 2 interventions were merged as one (Figure 3A).

NNgraph clustering (Figure 3B) resulted in 4 clusters, characterized by a different microbial signature and different concentrations of metabolic acids. Cluster 1 (7 participants) includes samples characterized by abundant bacteria belonging to the genus *Bifidobacterium*, (Supplementary Figure 3) along with elevated concentrations of isobutyric, isovaleric, and methylbutyric acids (Supplementary Figure 10). Cluster 2 (8 participants) consists of samples enriched by *Prevotella* members (Supplementary Figure 3), Cluster 3 (27 participants) regroups samples with low levels of observed richness and low concentrations of hexanoic acid (Supplementary Figure 10). Cluster 4 (25 participants) gathers participants with a higher concentration of hexanoic acid (Supplementary Figure 10). A total of 43 participants were defined as misclassified and were not assigned to any subpopulation. A significant increase in evenness indices (Shannon and Pielou) (*q* value < 0.001) and a significant decrease in dbp and divergence to median (*q* value <0.05) were observed in the *Prevotella* subpopulation (cluster 2) after the yogurt-rolled oats intervention, suggesting a homogenization of the gut microbial composition intra and between participants. Still, in cluster 2, a significant increase in fructosamine (*q* value < 0.001) was observed after the yogurt-only intervention (Supplementary Figure 11). None of the remaining clusters showed substantial changes in microbial composition, blood marker concentrations, or SCFA values after either intervention. A significant increase in evenness and a decrease in dominance detected in cluster 2 after the yogurt-rolled oats intervention were also observed when the 2 interventions were merged into one (Figure 3B). Additionally, a decrease in rarity (log modulo skewness) (*q* value < 0.001) and an increase in isobutyric, isovaleric, and methylbutyric acids concentrations (*q* value < 0.05) were detected in cluster 2, as well as an increase in evenness (Pielou) in cluster 4.

Overall, using 2 distinct clustering approaches allowed us to identify subpopulations within our study population based on different gut microbiota characteristics, such as level of diversity, gut microbial signature or SCFA concentrations. In the *Prevotella* subpopulation (cluster 2 NNgraph clustering), the most robust response was observed, showcasing a homogenization of the gut microbial composition intra and between participants and increased production of certain SCFAs after the yogurt-rolled oats intervention. On the contrary, among the participants who exhibited a low level of microbial diversity at the beginning of the study (cluster 1 kmeans), the concentrations of the same SCFAs slightly decreased after the 4-wk consumption of yogurt and rolled oats.

### Minor effects of yogurt and rolled oats consumption on the functional composition of the human gut microbiome

**FIGURE 4:**
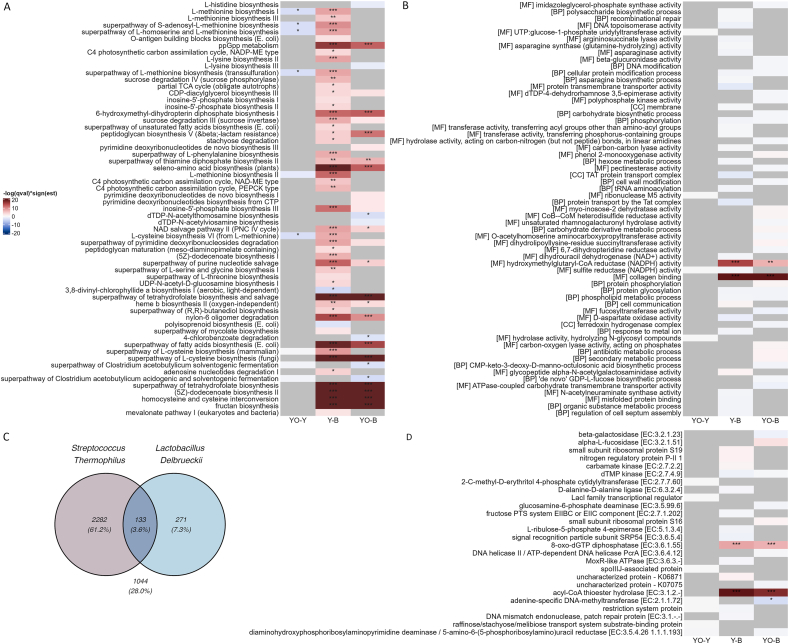
Effect of both interventions on the functional composition of the gut microbiome. Effect of each intervention on the relative abundance of pathways (A), GO terms (B), and KEGGs (D). Features with a *P* value < 0.01 are shown for visualization purposes. The color code (red increase, blue decrease) depicts the significance level of the estimated differences between yogurt vs. baseline (Y-B), yogurt-rolled oats vs. baseline (YO-B), yogurt-rolled oats vs. yogurt (YO-Y) based on *q* values adjusted for multiple testing. Statistical significance is indicated as: ∗*q* value < 0.05, ∗∗*q* value < 0.01, ∗∗∗*q* value < 0.001. (C) Venn diagram illustrating the proportion of significant genes originating from the yogurt-associated bacteria *Streptococcus thermophilus* and *Lactobacillus delbrueckii*, highlighting shared and unique genetic contributions from each species. Pathway, KEGG, and GO term IDs depicted in this figure can be found respectively in Supplementary Tables 2–4. GO, Gene Ontology; KEGG, Kyoto Encyclopedia of Genes and Genomes.

Of the 946,461 genes detected in over 10% of samples, 3492 (0.37%) showed a significant increase in relative abundance after the interventions (3017 after both, 347 only after yogurt, and 128 only after yogurt-rolled oats). Meanwhile, 238 genes (0.03%) showed a significant decrease (3 after both, 136 only after yogurt, and 99 only after yogurt-rolled oats). Of these 3730 genes with significant changes, 72% can be attributed to the 2 types of bacteria in the yogurt. In detail, 61.2% were specific for *Streptococcus thermophilus*, 7.3% for *Lactobacillus delbrueckii*, whereas 3.6% could be assigned to both bacterial species (Figure 4C).

Those 946,461 genes were subsequently filtered to focus on those with a minimum abundance of 1 CPM and regrouped into pathway, KEGG, and GO term families to better understand the effect of yogurt or yogurt and rolled oats consumption on the biological functions of the genes within the complex gut microbial community.

The pathway analysis revealed several significant changes after the interventions (Figure 4A). Most significant changes were detected after the yogurt intervention. Among them were several significantly enriched pathways associated with methionine biosynthesis. L-methionine biosynthesis II pathway had the highest counts assigned to *Bifidobacterium longum* and *Streptococcus thermophilus.* Inosine-5'-phosphate biosynthesis III pathway, significantly increased after the yogurt intervention, with *Streptococcus thermophilus* having the most counts assigned.

GO term analysis revealed 2 terms increasing after both interventions (Figure 4B), which are hydroxymethylglutaryl-CoA reductase (nicotinamide adenine dinucleotide phosphate) activity, which plays a crucial role in cholesterol biosynthesis [35], and collagen binding. Additionally, our analysis displayed 2 increasing KEGG pathways (Figure 4D), 8-oxo-dGTP diphosphatase [EC:3.6.1.55], which is involved in the repair of oxidized guanine residues in DNA [36,37] and acyl-CoA thioester hydrolase [EC:3.1.2.-].

In general, we observed few changes at the functional level. Most of the altered genes that increased after both interventions could be attributed to the enrichment of yogurt-derived bacteria in the host gut, with most signaling pathways being significantly enriched after the yogurt intervention. In summary, these results indicate that yogurt and rolled oats consumption does not result in major changes in the healthy gut microbiota at functional level.

## Discussion

In this intervention study, we investigated the effects of daily yogurt consumption (as a probiotic), as well as yogurt consumption in combination with rolled oats (as a combined probiotic and prebiotic), on the gut microbiota of healthy individuals. Our taxonomic data demonstrated a temporary enrichment of yogurt-derived bacteria, specifically *Streptococcus thermophilus* and *Lactobacillus delbrueckii*, during the intervention periods, which diminished after a washout phase. These findings align with an observational cohort study by Le Roy et al. [38], which investigated the link between gut microbiota and yogurt-related health benefits in >1000 predominantly female UK twins, as well as data from 1103 participants in the LifeLines-DEEP cohort, revealing an increased abundance of yogurt-associated species such as *S. thermophilus*. The functional analysis in our study mainly confirmed these observations. Only a very small fraction of the microbial metagenome was significantly altered. Most of these genes displayed a higher abundance after the interventions, which could be mainly attributed to the yogurt-derived bacteria. This indicates that although the study design effectively detects these transient changes, the overall gut microbiota composition remains stable. Further research is needed to understand how long yogurt bacteria can be detected after consumption ceases and to determine their potential role in the gut microbiome.

After the consumption of yogurt in combination with rolled oats, we observed a slight uniformization of the microbial composition at individual level and between participants. It is important to note that these changes mainly originated from the *Prevotella* subpopulation, which consisted of only 8 individuals who showed a very strong change. Within this subpopulation we also observed an increased abundance of SCFA that is in alignment with the established understanding of the genus *Prevotella*, known for its role in the fermentation of dietary fiber and the production of SCFAs [39,40].

These results emphasize that the individual microbial composition determines the microbiota’s ability to utilize the provided nutrients and consequently whether a nutritional intervention will be effective. This highlights the need for more personalized microbiota-based treatments, with dietary recommendations tailored to an individual’s unique microbiome instead of relying on a one-size-fits-all approach. Further studies should explore the impact of prebiotic and probiotic foods in greater detail, particularly by examining which nutritional interventions are most suitable for specific microbial compositions and how nutritional profiles could potentially preserve specific microbiome members [41].

A limitation of our study is that a formal sample size calculation was not performed. Microbiome data are complex, with high dimensionality, sparsity, strong overdispersion, and large interindividual variability, which violate the assumptions of standard power formulas [23]. In addition, the crossover design introduces further complexity, as sample size calculation for repeated or longitudinal measures requires specifying variance-covariance structures that are not well defined for microbiome data [24]. As a result, our sample size was chosen based on comparable human intervention studies rather than formal statistical power calculations. This limitation should be considered when interpreting the findings. An additional limitation is the nonpersonalized dosage of rolled oats and yogurt, which may have been excessive for some participants and insufficient for others. Although personalization based on baseline microbiome composition would likely provide more targeted and effective supplementation strategies, developing such an approach would require additional methodological work beyond the scope of this study. Future studies may explore this by integrating baseline microbiome profiles or simple physiological measures such as body weight to guide dosage adjustments. A further limitation of this study is that the intervention could not be blinded, as participants received commercially available products without a placebo or control foods. The absence of participant and investigator blinding may introduce performance or detection bias and should be taken into account when interpreting the results. Future studies could reduce such bias by using control foods with comparable texture and taste, such as yogurt or oatmeal-based placebos, to enable participant and investigator blinding. Furthermore, the study was conducted in healthy individuals to establish baseline effects under controlled physiological conditions and to minimize confounding factors related to disease or medication. However, this limits the generalizability of the findings to clinical populations. Future studies should confirm these findings in relevant patient groups. Finally, a strict intention-to-treat analysis could not be performed, as 9 of the 119 randomly divided participants withdrew before completing all study phases. Although this represents only 7.6% of the total sample and is not expected to substantially affect the overall findings, it should be taken into consideration when interpreting the results.

Overall, our study suggests that personalized dietary recommendations may be the most effective approach for optimizing dietary interventions or treatments aimed at improving the gut microbiome. Studies involving a personalized approach could provide deeper insights into the nuanced effects of diet on gut health [42,43]. Future research should consider the individualized responses to dietary interventions, especially given the differences in gut microbiota stability between healthy individuals and patients with conditions such as inflammatory bowel disease or *Clostridium difficile* infections. It would be beneficial to compare the effects of yogurt and rolled oats consumption in healthy individuals compared with patients to refine dietary guidelines. Additionally, further research is needed to explore the role of prebiotics and probiotics in maintaining a balanced gut microbiome in healthy individuals and their potential benefits for those with gut dysbiosis. Previous studies have shown that prebiotics and probiotics can be beneficial, particularly in precision medicine applications aimed at correcting imbalances in the gut microbiome [2,44]. Additionally, further research is needed to explore the role of prebiotics and probiotics in maintaining a balanced gut microbiome in healthy individuals and their potential benefits for those with gut dysbiosis.

In general, defining a “healthy gut microbiota” remains challenging [45]. Still, it is generally believed that greater microbial diversity correlates with resilience against invasive pathogens [1], and more efficient nutrient utilization, contributing to overall health. Microbial communities are crucial in driving key ecosystem processes across diverse habitats [46]. Their resilience, or the ability to withstand and recover from disturbances, is an important determinant of ecosystem functioning and stability [47,48]. Higher diversity and functional redundancy within microbial communities can enhance resilience by providing a buffer against disturbances. Diverse communities are more likely to contain taxa capable of tolerating or adapting to changing conditions [49].

Despite the temporary presence of yogurt-derived bacteria in stool samples during yogurt consumption over the course of the interventions, our study revealed no lasting effects on the participants’ overall gut microbial composition or health parameters. The transient nature of these changes suggests that the yogurt bacteria likely pass through the digestive system without establishing a long-term presence in the gut microbiota. This indicates that the gut microbiome of the healthy participants in our study is resilient and can protect the host from transient alterations in diet and possibly other environmental stimuli.

In summary, the results of our study suggest that a healthy person’s gut microbiome is resilient to temporary dietary interventions such as yogurt and rolled oats consumption. It also shows that the individual composition of a microbial community determines whether a nutritional intervention has an effect and whether the microbiota can utilize the provided nutrients. This highlights the need for more personalized approaches with dietary recommendations tailored to each individual’s unique microbiome.

In conclusion, a healthy person’s gut microbiome is mainly resilient to temporary dietary interventions such as yogurt and rolled oats consumption. Yogurt intake temporarily increases yogurt-derived bacteria in the human gut microbiome, whereas additional consumption of rolled oats alters the microbial composition in those with a Prevotella-dominant gut microbiota. These results highlight that the effectiveness of a nutritional intervention depends on individual microbial composition, emphasizing the need for personalized microbiota-based treatments and tailored dietary recommendations.

## Author contributions

The author’s responsibilities were as follows – KT, KBM: conceived and designed the study; KT: conducted research; KT, VS, KDH, TS, KBM: defined the methodology; VS, KDH: curated the data; KDH: performed the bioinformatic analysis and VS the statistical analysis; KT, VS: wrote the paper; KBM: provided resources and supervised the work; and all authors: read, commented, and approved the final manuscript.

## Data availability

The metagenomic sequencing data generated during the previous study are available in the NCBI Sequence Read Archive under the accession number PRJNA1258884 (https://www.ncbi.nlm.nih.gov/bioproject/PRJNA1258884/). Processed short-chain fatty acid concentration data have been deposited in Zenodo and are available at https://doi.org/10.5281/zenodo.15363886. Additional datasets, including clinical blood markers and study results, as well as all R scripts used for statistical analysis and figure generation, are available on GitHub (https://github.com/vstanislas/IPE_yogurt_rolledOat_microbiome).

## Funding

The authors reported no funding received for this study.

## Conflict of interest

The authors report no conflicts of interest.
