# Supplementary material for: Impact of Yogurt and Rolled Oats Consumption on the Gut Microbiome: A Randomized Crossover Study Displaying Individual Responses and General Resilience
Source: J Nutr. 2026 Feb 11;156(4):101408. doi: 10.1016/j.tjnut.2026.101408 (PMC13084677; doi:10.1016/j.tjnut.2026.101408)
Supplement: Multimedia component 1 [file mmc1.docx]

**Impact of Yogurt and Rolled Oats on the Gut Microbiome: A Randomized Crossover Study displaying Individual Responses and General Resilience**

Kerstin Thriene^1,2^, Virginie Stanislas^1^, Kun D. Huang^3^, Till Strowig^3,4^, and Karin B. Michels^1,*^

^1^Institute for Prevention and Cancer Epidemiology, Faculty of Medicine and Medical Center, University of Freiburg, 79110 Freiburg, Germany

^2^Institute of Microbiology, Friedrich Schiller University, Jena, Germany.

^3^Department of Microbial Immune Regulation, Helmholtz Center for Infection Research, 38124 Braunschweig, Germany

^4^Centre for Individualised Infection Medicine (CiiM), a joint venture between the Helmholtz-Centre for Infection Research (HZI) and the Hannover Medical School (MHH), Hannover, Germany

**^*^Corresponding author**: Karin B. Michels; Institute for Prevention and Cancer Epidemiology; Faculty of Medicine and Medical Center; University of Freiburg; Elsässer Straße 2; 79110 Freiburg, Germany.

E-mail: [tumorepidemiologie@uniklinik-freiburg.de](mailto:tumorepidemiologie@uniklinik-freiburg.de)

**Supplementary material**

**SUPPLEMENTARY METHODS**

We provide below additional explanation of the regression model to aid comprehension.

Example of variable coding for 3 participants:

| Participant | Group | Intervention | B | T | C1 | C2 | P |
| --- | --- | --- | --- | --- | --- | --- | --- |
| S_11_ | A | Baseline 1 | 0 | 0 | 0 | 0 | 0 |
| S_12_ | A | Yogurt | 1 | 0 | 0 | 0 | 0 |
| S_13_ | A | Baseline 2 | 0 | 0 | 1 | 0 | 1 |
| S_14_ | A | Yogurt + Oat | 1 | 1 | 1 | 0 | 1 |
| S_21_ | B | Baseline 1 | 0 | 0 | 0 | 0 | 0 |
| S_22_ | B | Yogurt + Oat | 1 | 1 | 0 | 0 | 0 |
| S_23_ | B | Baseline 2 | 0 | 0 | 0 | 1 | 1 |
| S_24_ | B | Yogurt | 1 | 0 | 0 | 1 | 1 |
| S_31_ | A | Baseline 1 | 0 | 0 | 0 | 0 | 0 |
| S_32_ | A | Yogurt | 1 | 0 | 0 | 0 | 0 |
| S_33_ | A | Baseline 2 | 0 | 0 | 1 | 0 | 1 |
| S_34_ | A | Yogurt + Oat | 1 | 1 | 1 | 0 | 1 |

$B_{\left\{ ijk \right\}}$ : 0 for baseline or 1 for other

$P_{\left\{ ijk \right\}}$: 0 for 1^st^ period and 1 for 2nd period (1st period = Phase II, 2nd period = Phase IV)

$T_{\left\{ ijk \right\}}$ : 0 or 1 if after yogurt rolled oats

$C1_{\left\{ ijk \right\}}$ : 0 or 1 for carry-over of yogurt from 1st period into 2nd period

$C2_{\left\{ ijk \right\}}$ : 0 or 1 for carry-over of yogurt rolled oats from 1st period into 2nd period

**Model 1:**

$$\boldsymbol{Y}_{\left\{ \boldsymbol{ijk} \right\}}\boldsymbol{=\mu+}\boldsymbol{\beta}_{\boldsymbol{1}}\boldsymbol{B}_{\left\{ \boldsymbol{ijk} \right\}}\boldsymbol{+}\boldsymbol{\beta}_{\boldsymbol{2}}\boldsymbol{T}_{\left\{ \boldsymbol{ijk} \right\}}\boldsymbol{+}\boldsymbol{\beta}_{\boldsymbol{3}}\boldsymbol{C}\boldsymbol{1}_{\left\{ \boldsymbol{ijk} \right\}}\boldsymbol{+}\boldsymbol{\beta}_{\boldsymbol{4}}\boldsymbol{C}\boldsymbol{2}_{\left\{ \boldsymbol{ijk} \right\}}\boldsymbol{+}\boldsymbol{s}_{\left\{ \boldsymbol{ik} \right\}}\boldsymbol{+}\boldsymbol{\epsilon}_{\left\{ \boldsymbol{ijk} \right\}}$$

Derivate sub-models:

$\boldsymbol{\mu}$ = 1^st^ baseline

$\boldsymbol{\mu+}\boldsymbol{\beta}_{\boldsymbol{3}}$ = 2^nd^ baseline following Yogurt

$\boldsymbol{\mu+}\boldsymbol{\beta}_{\boldsymbol{4}}$ = 2^nd^ baseline following Yogurt + Oats

$\boldsymbol{\mu+}\boldsymbol{\beta}_{\boldsymbol{1}}$ = After Yogurt in Group A

$\boldsymbol{\mu+}\boldsymbol{\beta}_{\boldsymbol{1}}\boldsymbol{+}\boldsymbol{\beta}_{\boldsymbol{4}}$ = After Yogurt in Group B

$\boldsymbol{\mu+}\boldsymbol{\beta}_{\boldsymbol{1}}\boldsymbol{+}\boldsymbol{\beta}_{\boldsymbol{2}}\boldsymbol{+}\boldsymbol{\beta}_{\boldsymbol{3}}$ = After Yogurt + Oats in Group A

$\boldsymbol{\mu+}\boldsymbol{\beta}_{\boldsymbol{1}}\boldsymbol{+}\boldsymbol{\beta}_{\boldsymbol{2}}$ = After Yogurt + Oats in Group B

Interpretation of the coefficients:

$\boldsymbol{\beta}_{\boldsymbol{1}}$: Yogurt vs Baseline

$\boldsymbol{\beta}_{\boldsymbol{1}}+ \boldsymbol{\beta}_{\boldsymbol{2}}$: Yogurt + Oats vs Baseline

$\boldsymbol{\beta}_{\boldsymbol{2}}+ \boldsymbol{\beta}_{\boldsymbol{3}}$: Yogurt + Oats vs Yogurt in group A

$\boldsymbol{\beta}_{\boldsymbol{2}}- \boldsymbol{\beta}_{\boldsymbol{4}}$: Yogurt + Oats vs Yogurt in group B

$\boldsymbol{\beta}_{\boldsymbol{2}}$**: (Yogurt + Oats vs Baseline) vs (Yogurt vs Baseline)**

**Model 2:**

$$\boldsymbol{Y}_{\left\{ \boldsymbol{ijk} \right\}}\boldsymbol{=\mu+}\boldsymbol{\beta}_{\boldsymbol{1}}\boldsymbol{B}_{\left\{ \boldsymbol{ijk} \right\}}\boldsymbol{+}\boldsymbol{\beta}_{\boldsymbol{2}}\boldsymbol{P}_{\left\{ \boldsymbol{ijk} \right\}}\boldsymbol{+}\boldsymbol{s}_{\left\{ \boldsymbol{ik} \right\}}\boldsymbol{+}\boldsymbol{\epsilon}_{\left\{ \boldsymbol{ijk} \right\}}$$

Derivate sub-models:

$\boldsymbol{\mu}$ = Baseline in Period 1

$\boldsymbol{\mu+}\boldsymbol{\beta}_{\boldsymbol{2}}$ = Baseline in Period 2

$\boldsymbol{\mu+}\boldsymbol{\beta}_{\boldsymbol{1}}$ = After intervention Period 1

$\boldsymbol{\mu+}\boldsymbol{\beta}_{\boldsymbol{1}}\boldsymbol{+}\boldsymbol{\beta}_{\boldsymbol{2}}$ = After intervention Period 2

Interpretation of the coefficients:

$\boldsymbol{\beta}_{\boldsymbol{1}}$**: After intervention vs Baseline**

$\boldsymbol{\beta}_{\boldsymbol{2}}$: Period 2 vs Period 1

**SUPPLEMENTARY FIGURES**

**
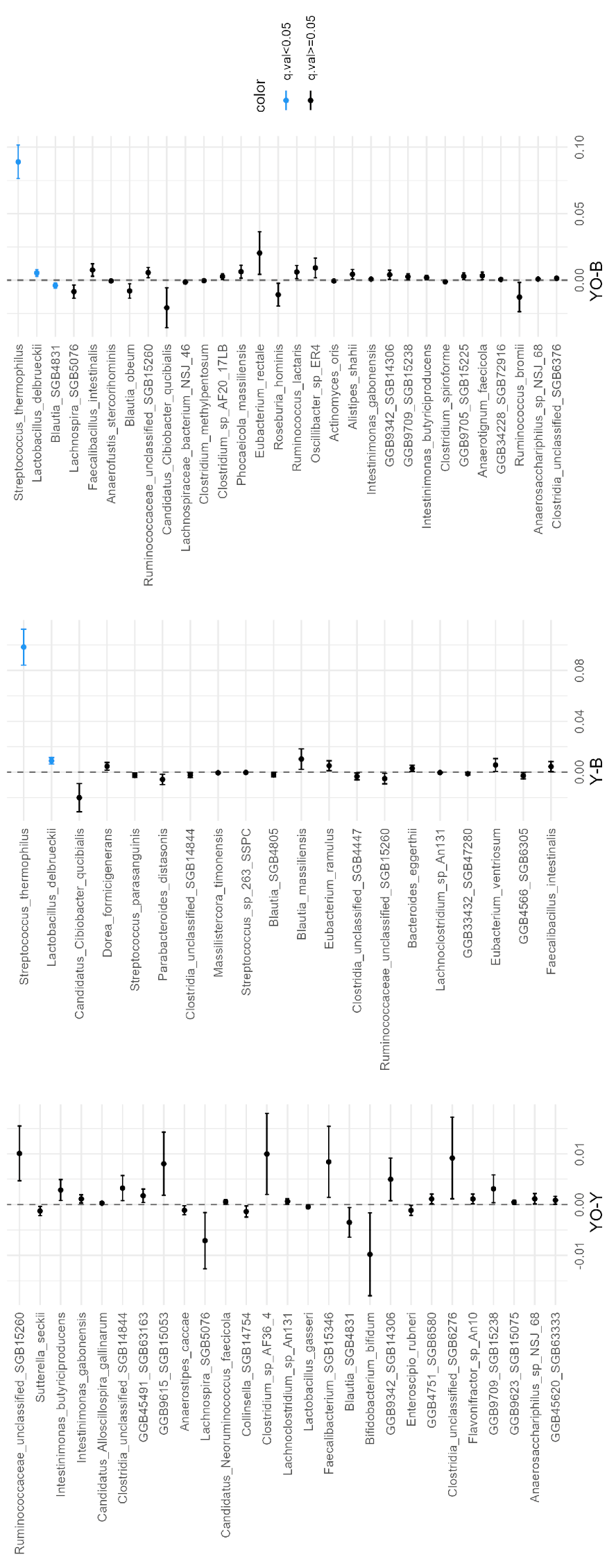
**

**Supplementary Figure 1: Effect sizes (estimates + confidence intervals) of the top detected species depicted in Figure 2.**  Species are ordered by level of significance for each comparison: yogurt vs. baseline (Y-B), yogurt rolled oats vs. baseline (YO-B), yogurt rolled oats vs. yogurt (YO-Y).

| 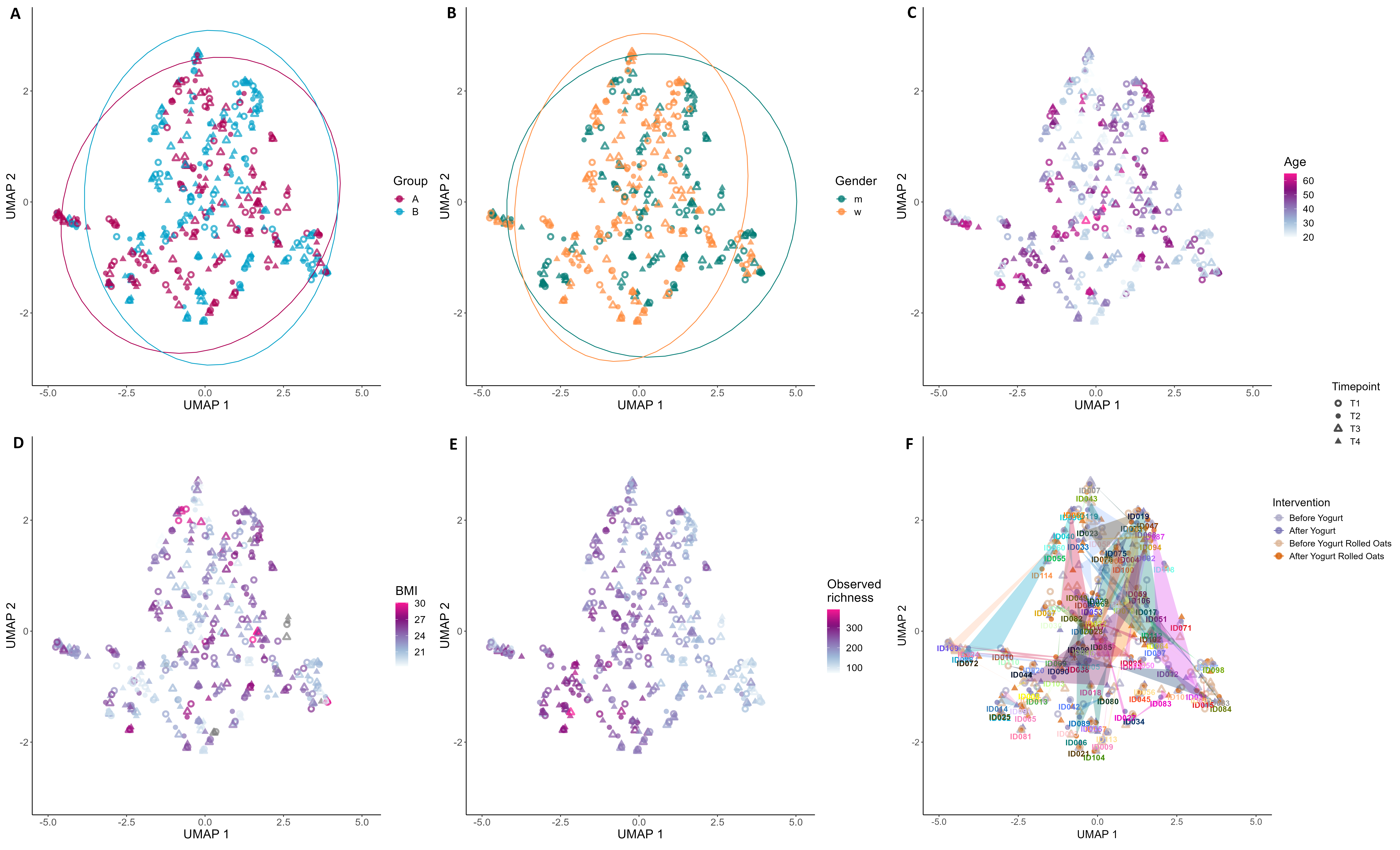 |
| --- |
| **Supplementary Figure 2:** **UMAP representations of the gut microbial composition.** Sample points are colored by intervention group, participant characteristics and observed richness with 95% data ellipse for each categorical subgroup. In the last UMAP plot, the samples that belong to the same participant are connected to each other, forming a polygon. |

| 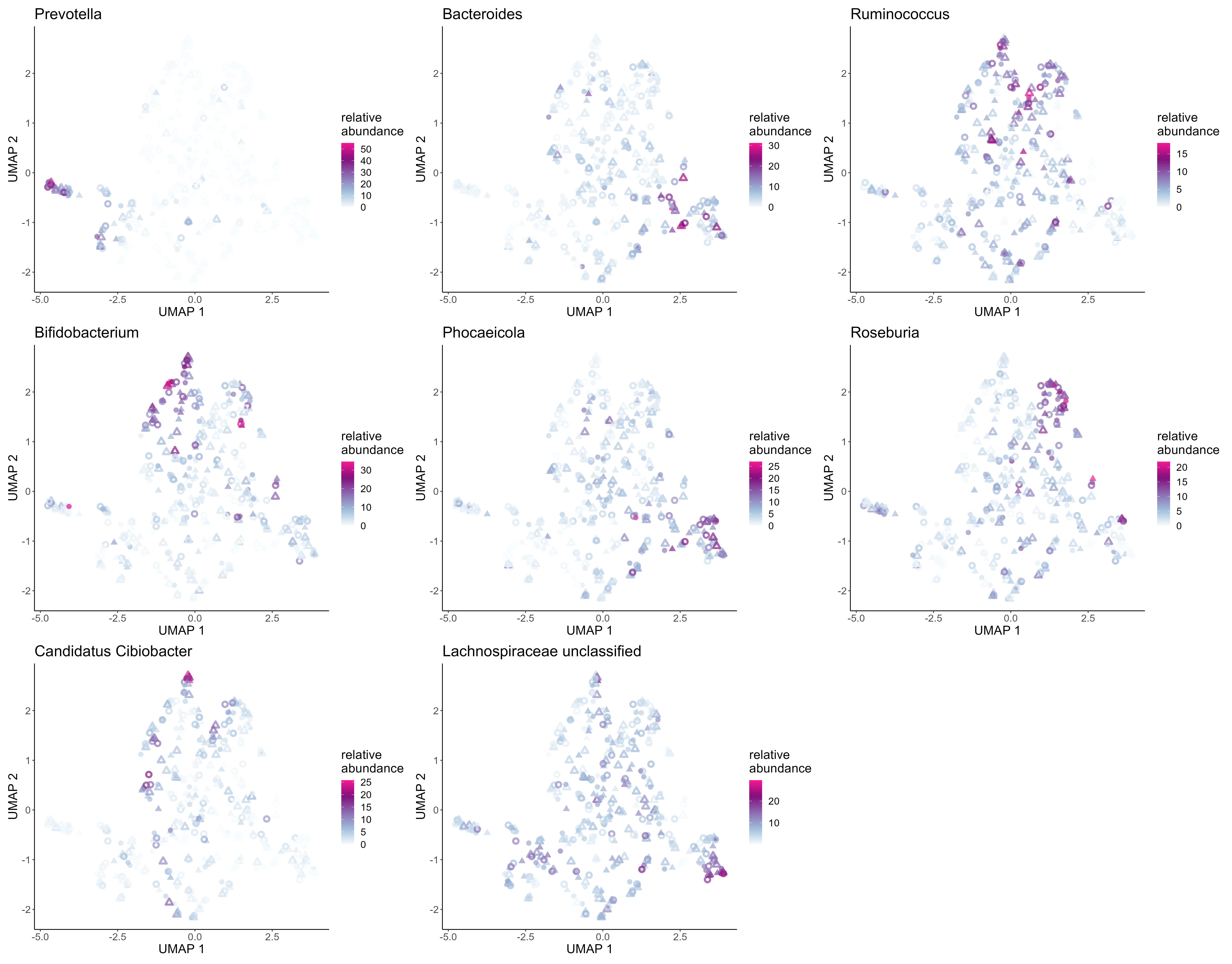 |
| --- |
| **Supplementary Figure 3:** **UMAP representation of the gut microbial composition.** Sample points are colored by relative abundance of a set of discriminating species. |

| **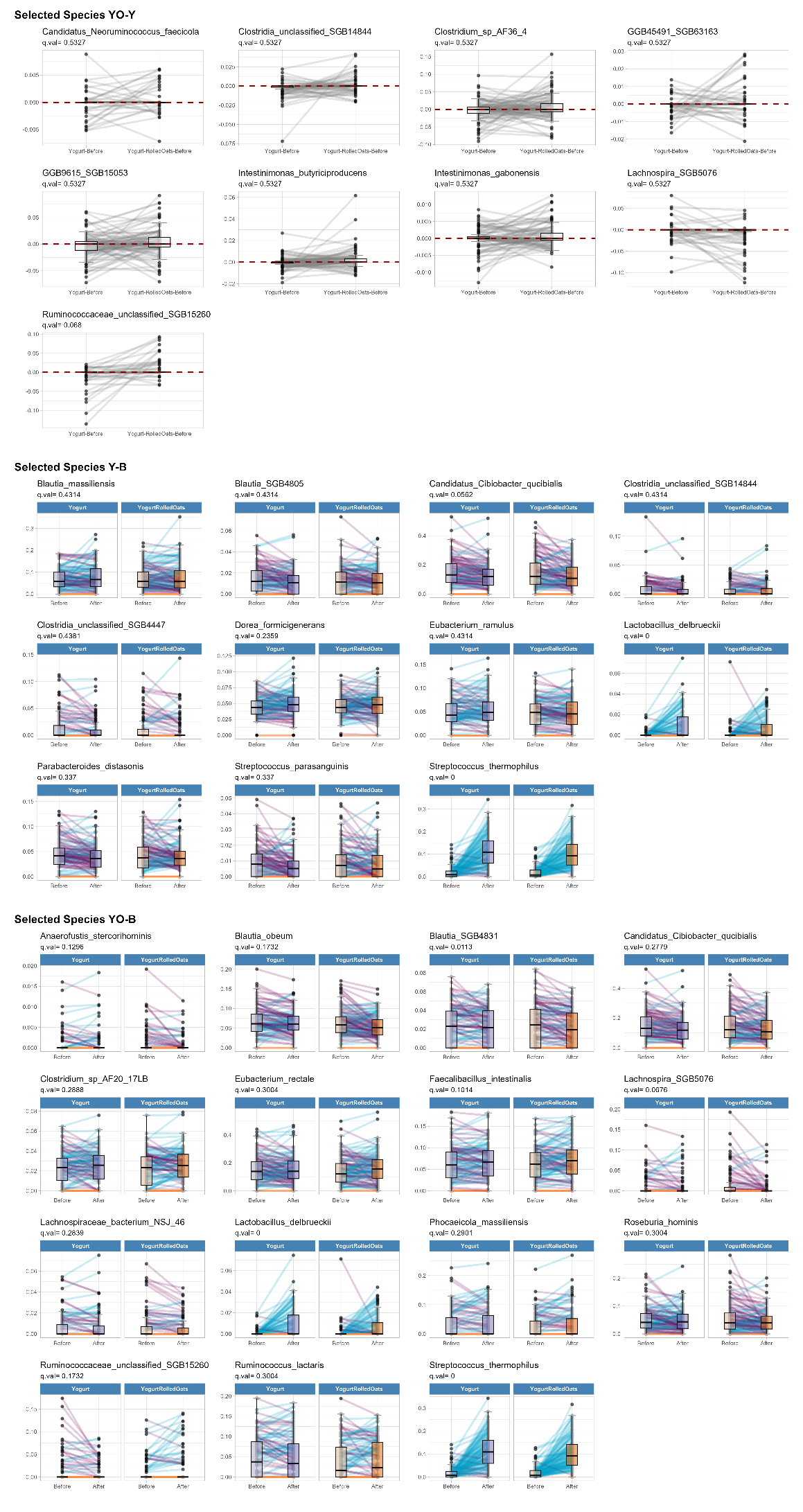** |
| --- |
| **Supplementary Figure 4:** **Changes in relative abundance of the species depicted in Figure 2D before and after each intervention.** Plots corresponding to the selected species YO-Y (yogurt rolled oats vs. yogurt) display the difference in change from baseline between the two interventions. Each point represents the change of relative abundance after vs. before intervention. |

| 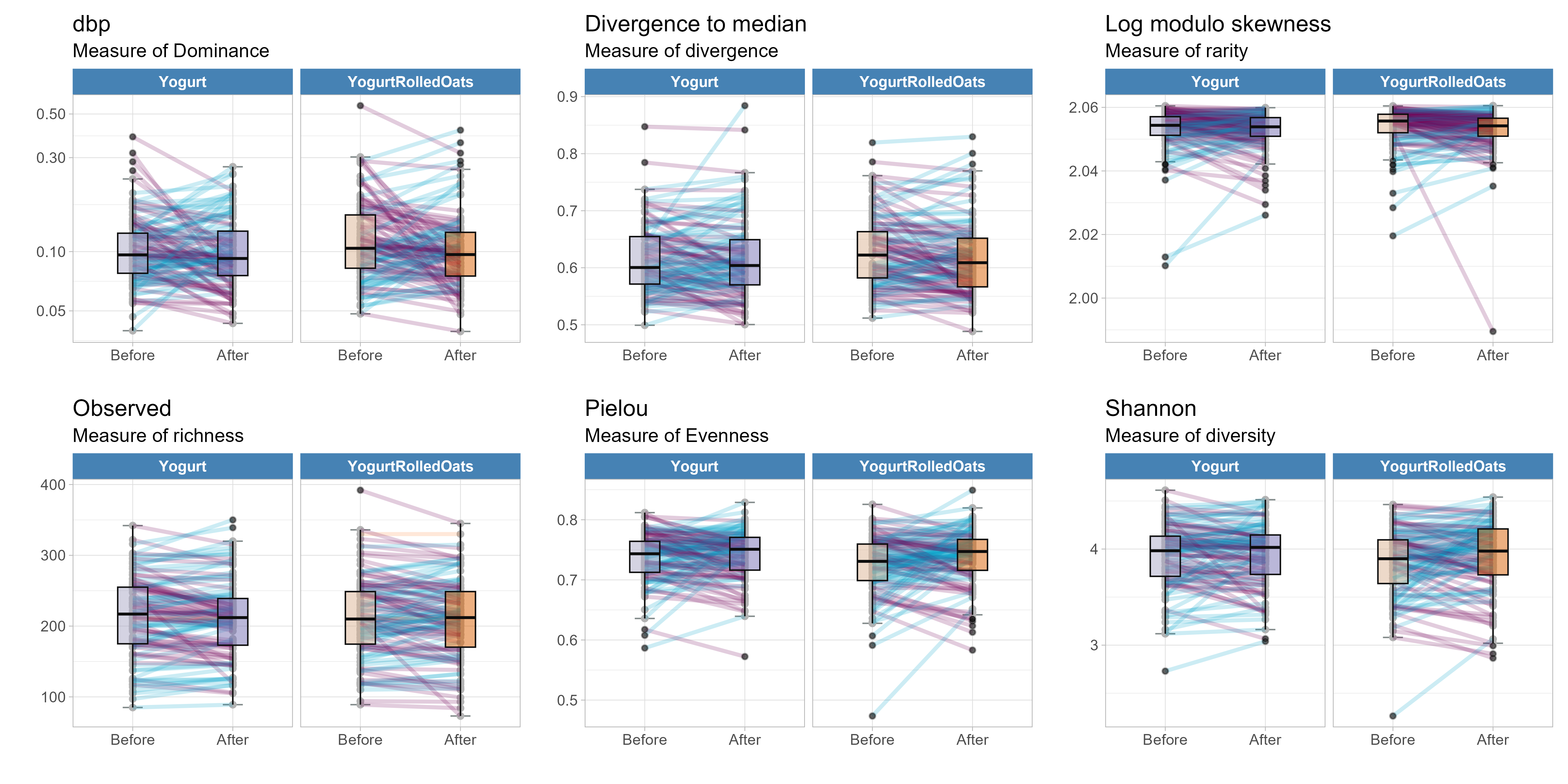 |
| --- |
| **Supplementary Figure 5:** **Changes in alpha diversity before and after each intervention.** |

| **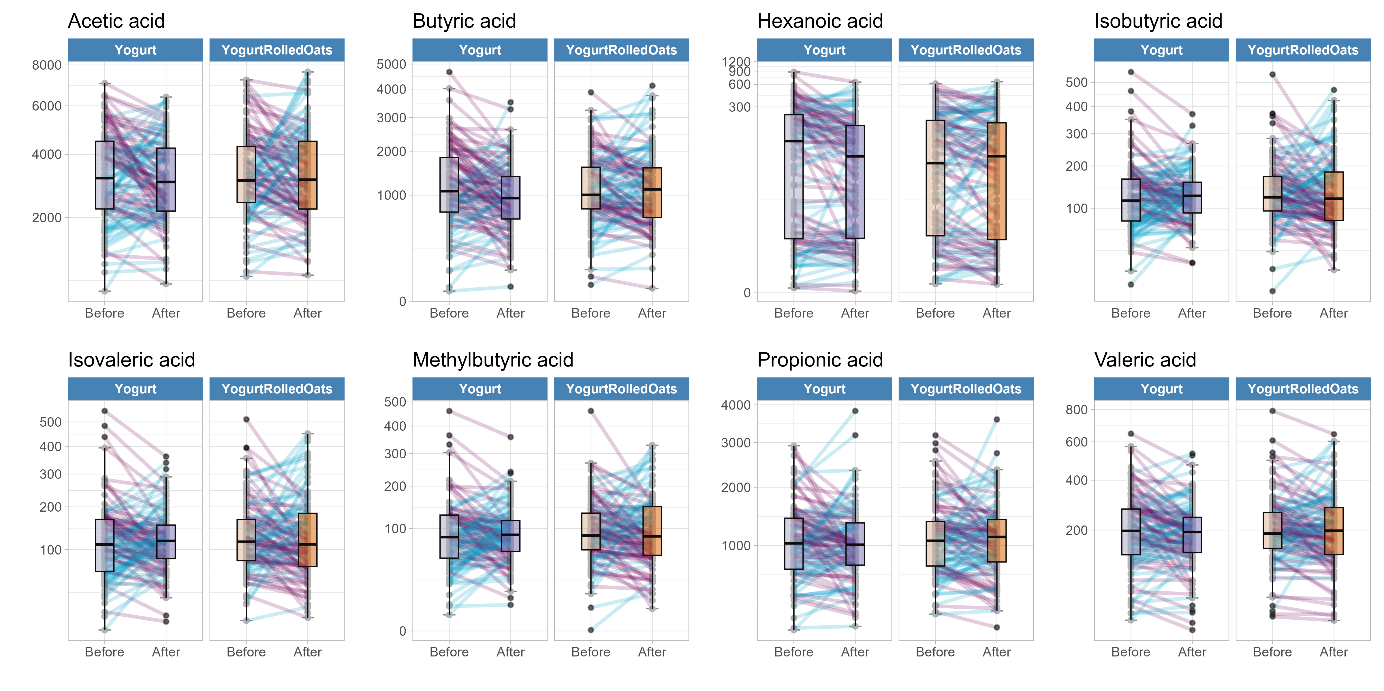** |
| --- |
| **Supplementary Figure 6: Changes in metabolite concentration before and after each intervention.** |

| **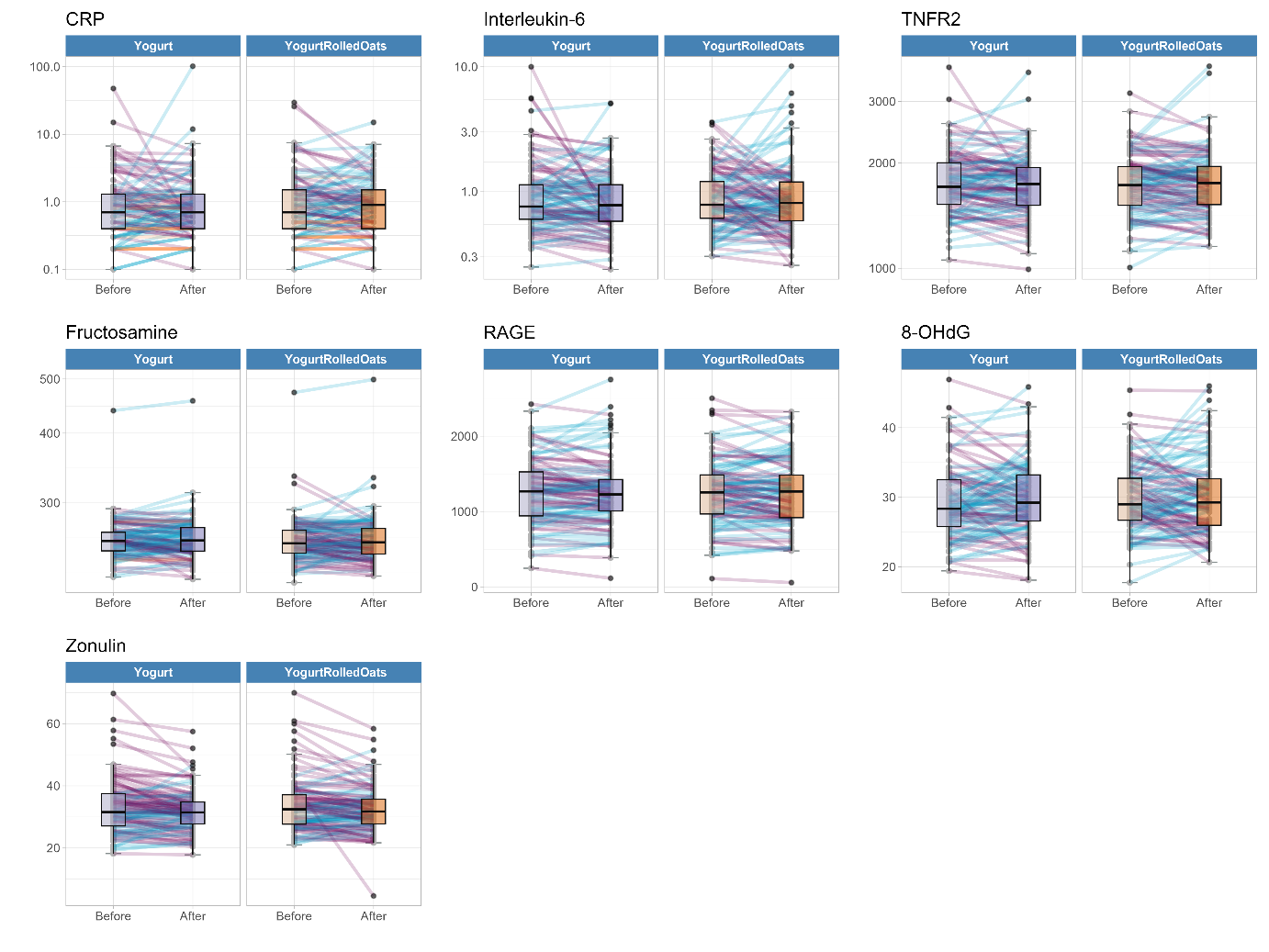** |
| --- |
| **Supplementary Figure 7:** **Changes in blood marker concentration before and** **after each intervention.** |

| **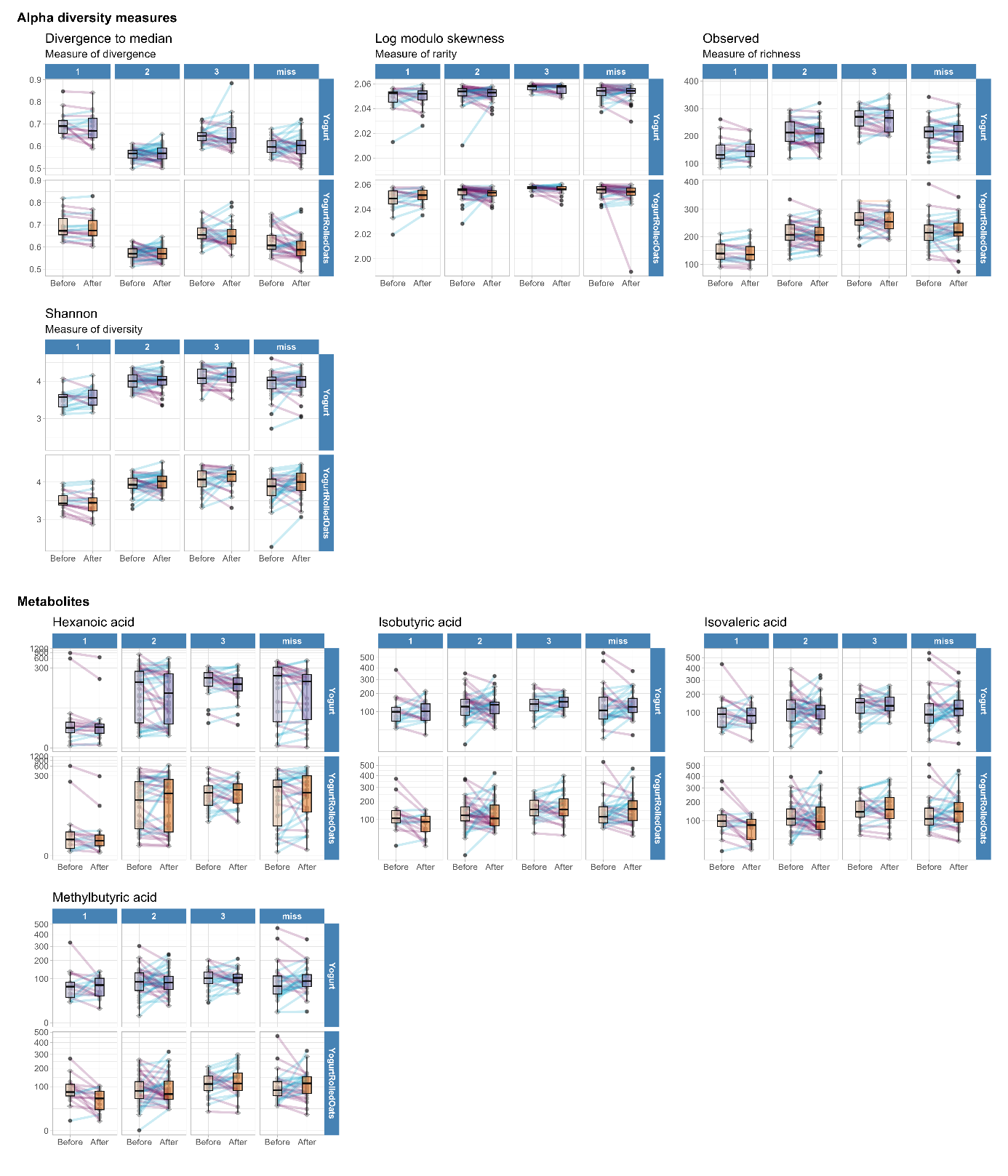** |
| --- |
| **Supplementary Figure 8:** **Changes before and after the intervention in each kmeans cluster for a set of alpha diversity measures and metabolites.** These alpha diversity measures and metabolites were selected either because they allow to describe the difference between the kmeans clusters or because they were found to be significantly changed in at least one of the interventions in one of the clusters (Figure 3, Sup Figure 7). |

| **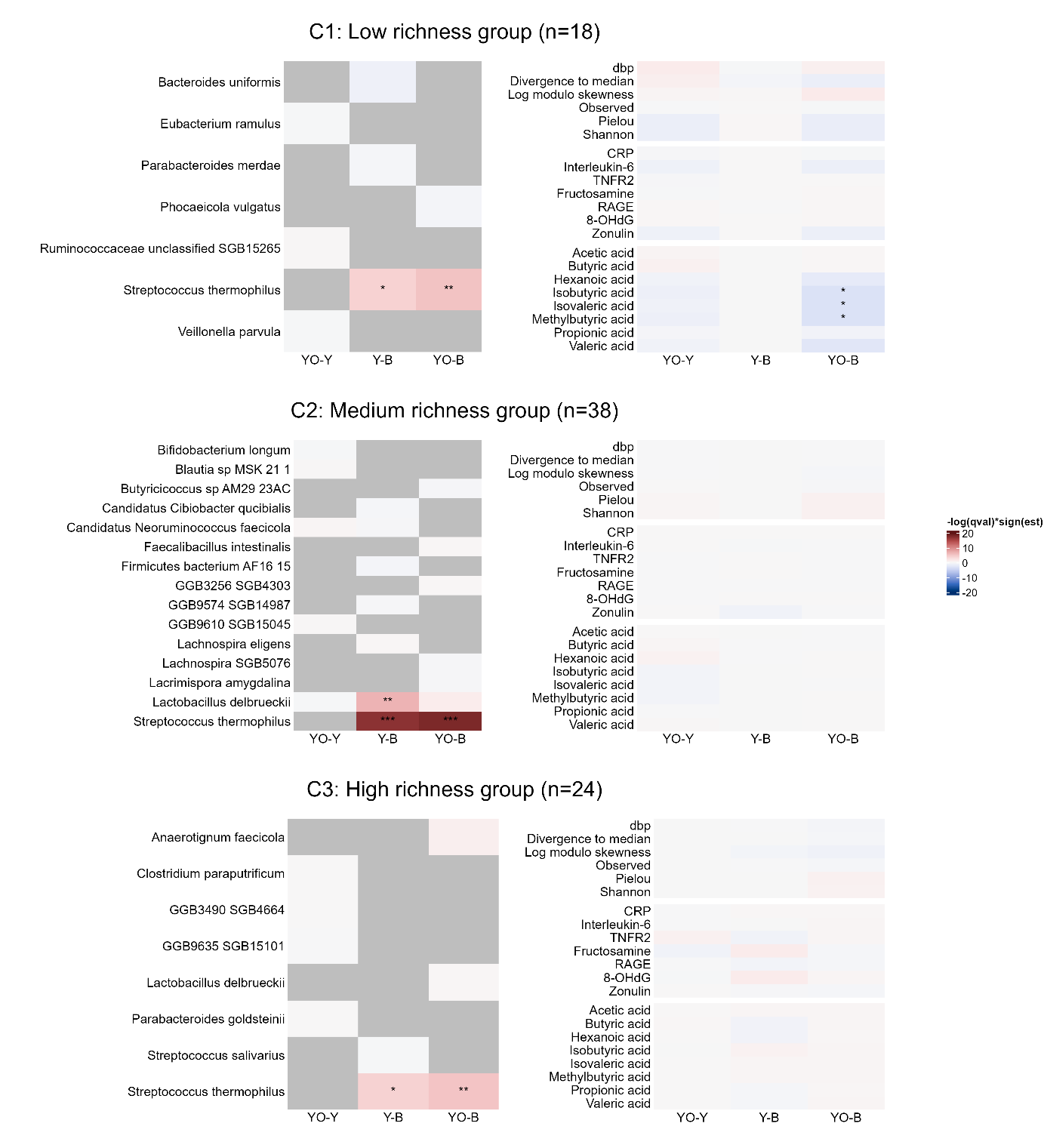** |
| --- |
| **Supplement****ary Figure 9:** **Effect of each intervention on alpha diversity measures, blood markers, metabolite concentrations and relative abundance of each species in each kmeans cluster.** Species with a p-value < 0.015 are shown for visualization purposes. The color code (red increase, blue decrease) depicts the significance level of the estimated differences between: yogurt vs. baseline (Y-B), yogurt rolled oats vs. baseline (YO-B), yogurt rolled oats vs. yogurt (YO-Y) based on q-values adjusted for multiple testing. Statistical significance is indicated as: * q-value < 0.05, ** q-value < 0.01, *** q-value <0.001. |

| **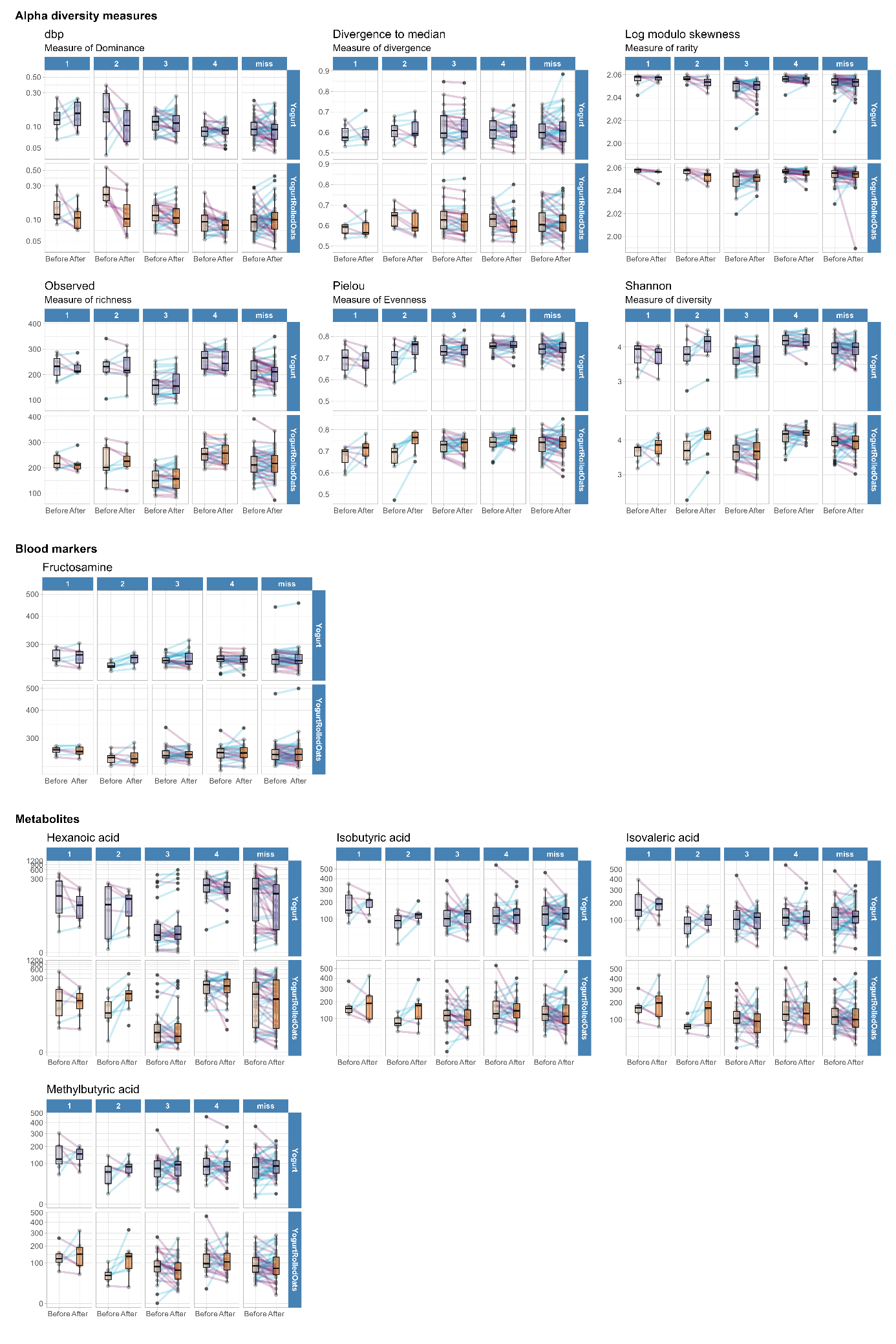** |
| --- |
| **Supplementary Figure 10:** **Changes before and after the intervention in each NNgraph cluster for a set of alpha diversity measures, blood markers and metabolites.** These alpha diversity measures, blood markers and metabolites were selected either because they allow to describe the difference between the NNgraph clusters or because they were found to be significantly changed in at least one of the interventions in one of the clusters (Figure 3, Sup Figure 8). |

| **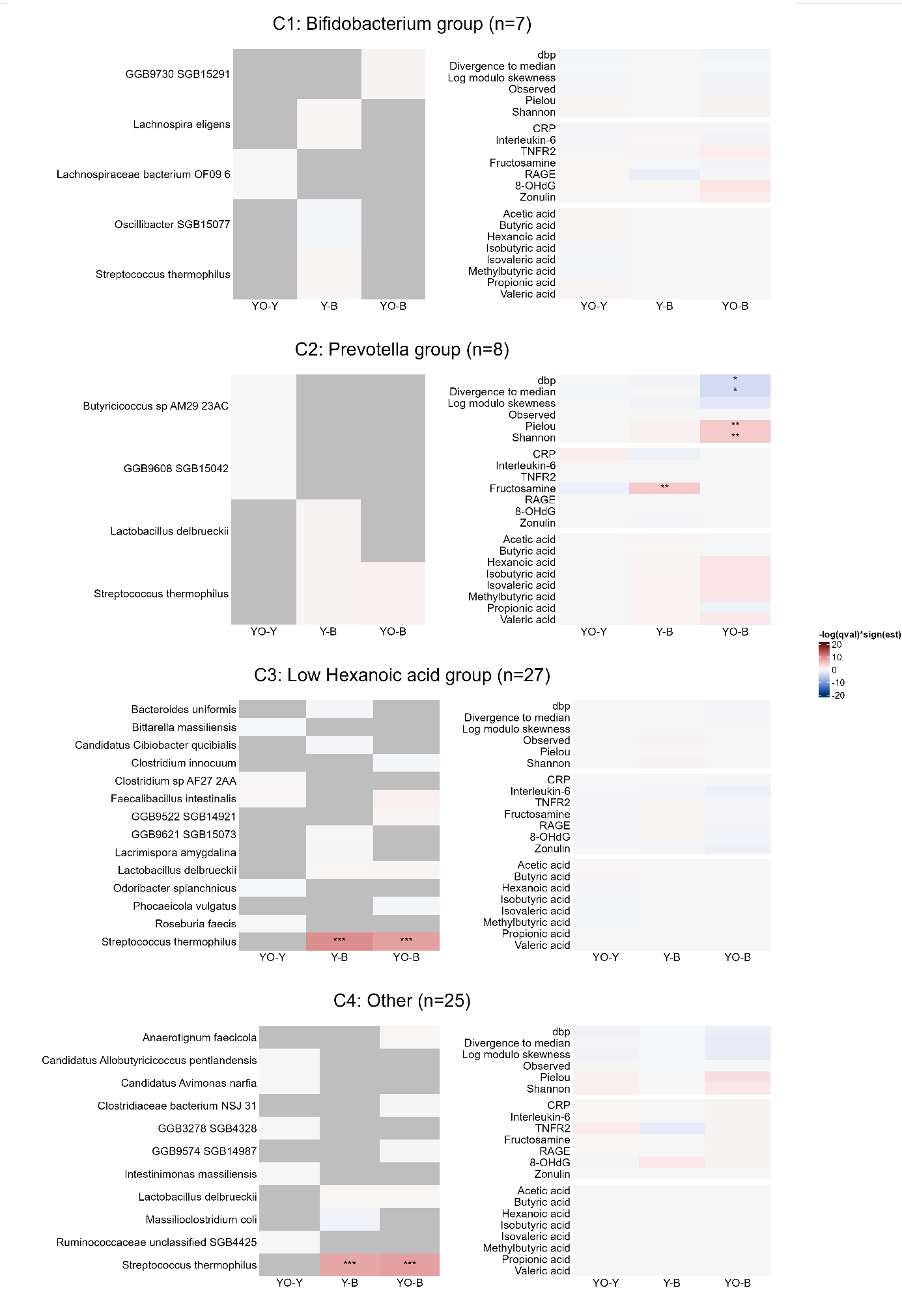** |
| --- |
| **Supplementary Figure 11: Effect of each intervention on alpha diversity measures, blood markers, metabolite concentrations and relative abundance of each species in each NNgraph cluster.** Species with a p-value < 0.015 are shown for visualization purposes. The color code (red increase, blue decrease) depicts the significance level of the estimated differences between: yogurt vs. baseline (Y-B), yogurt rolled oats vs. baseline (YO-B), yogurt rolled oats vs. yogurt (YO-Y) based on q-values adjusted for multiple testing. Statistical significance is indicated as: * q-value < 0.05, ** q-value < 0.01, *** q-value <0.001. |

**SUPPLEMENTARY TABLES**

**Supplementary Table 1: Results of PERMANOVA analysis at baseline.**

|  | ***Df*** | ***Sums of Squares*** | ***R^2^*** | ***F-value*** | ***P-value*** | ***Significance*** |
| --- | --- | --- | --- | --- | --- | --- |
| Group | 1 | 0.2202 | 0.0089 | 1.1024 | 0.2791 |  |
| BMI | 1 | 0.1925 | 0.0078 | 0.9638 | 0.5102 |  |
| Age | 1 | 0.2422 | 0.0098 | 1.2124 | 0.1634 |  |
| Gender | 1 | 0.2634 | 0.0106 | 1.3187 | 0.1034 |  |
| Observed richness | 1 | 1.2981 | 0.0523 | 6.4989 | 0.0001 | *** |
| Prevotella | 1 | 1.0014 | 0.0404 | 5.0136 | 0.0001 | *** |
| Bacteroides | 1 | 0.7478 | 0.0301 | 3.7439 | 0.0001 | *** |
| Ruminococcus | 1 | 0.5389 | 0.0217 | 2.6978 | 0.0001 | *** |
| Residual | 97 | 19.375 | 0.781 |  |  |  |
| Total | 105 | 24.8076 | 1 |  |  |  |

Significance levels p-values: * <0.05, ** <0.01, *** <0.001

Df = degree of freedom; BMI = Body mass index; *R^2^* = coefficient of determination.

**Supplementary Table 2: Pathways IDs and their description as depicted in Figure 4.**

| **Pathway** | **Description** |
| --- | --- |
| HISTSYN PWY | L-histidine biosynthesis |
| HOMOSER METSYN PWY | L-methionine biosynthesis I |
| HSERMETANA PWY | L-methionine biosynthesis III |
| MET SAM PWY | superpathway of S-adenosyl-L-methionine biosynthesis |
| METSYN PWY | superpathway of L-homoserine and L-methionine biosynthesis |
| OANTIGEN PWY | O-antigen building blocks biosynthesis (E. coli) |
| PPGPPMET PWY | ppGpp metabolism |
| PWY 241 | C4 photosynthetic carbon assimilation cycle, NADP-ME type |
| PWY 2941 | L-lysine biosynthesis II |
| PWY 2942 | L-lysine biosynthesis III |
| PWY 5347 | superpathway of L-methionine biosynthesis (transsulfuration) |
| PWY 5384 | sucrose degradation IV (sucrose phosphorylase) |
| PWY 5913 | partial TCA cycle (obligate autotrophs) |
| PWY 5981 | CDP-diacylglycerol biosynthesis III |
| PWY 6123 | inosine-5'-phosphate biosynthesis I |
| PWY 6124 | inosine-5'-phosphate biosynthesis II |
| PWY 6147 | 6-hydroxymethyl-dihydropterin diphosphate biosynthesis I |
| PWY 621 | sucrose degradation III (sucrose invertase) |
| PWY 6284 | superpathway of unsaturated fatty acids biosynthesis (E. coli) |
| PWY 6470 | peptidoglycan biosynthesis V (&beta;-lactam resistance) |
| PWY 6527 | stachyose degradation |
| PWY 6545 | pyrimidine deoxyribonucleotides de novo biosynthesis III |
| PWY 6628 | superpathway of L-phenylalanine biosynthesis |
| PWY 6895 | superpathway of thiamine diphosphate biosynthesis II |
| PWY 6936 | seleno-amino acid biosynthesis (plants) |
| PWY 702 | L-methionine biosynthesis II |
| PWY 7115 | C4 photosynthetic carbon assimilation cycle, NAD-ME type |
| PWY 7117 | C4 photosynthetic carbon assimilation cycle, PEPCK type |
| PWY 7184 | pyrimidine deoxyribonucleotides de novo biosynthesis I |
| PWY 7210 | pyrimidine deoxyribonucleotides biosynthesis from CTP |
| PWY 7234 | inosine-5'-phosphate biosynthesis III |
| PWY 7315 | dTDP-N-acetylthomosamine biosynthesis |
| PWY 7316 | dTDP-N-acetylviosamine biosynthesis |
| PWY 7761 | NAD salvage pathway II (PNC IV cycle) |
| PWY I9 | L-cysteine biosynthesis VI (from L-methionine) |
| PWY0 1298 | superpathway of pyrimidine deoxyribonucleosides degradation |
| PWY0 1586 | peptidoglycan maturation (meso-diaminopimelate containing) |
| PWY0 862 | (5Z)-dodecenoate biosynthesis I |
| PWY66 409 | superpathway of purine nucleotide salvage |
| SER GLYSYN PWY | superpathway of L-serine and glycine biosynthesis I |
| THRESYN PWY | superpathway of L-threonine biosynthesis |
| UDPNAGSYN PWY | UDP-N-acetyl-D-glucosamine biosynthesis I |
| CHLOROPHYLL SYN | 3,8-divinyl-chlorophyllide a biosynthesis I (aerobic, light-dependent) |
| FOLSYN PWY | superpathway of tetrahydrofolate biosynthesis and salvage |
| HEMESYN2 PWY | heme b biosynthesis II (oxygen-independent) |
| P125 PWY | superpathway of (R,R)-butanediol biosynthesis |
| P621 PWY | nylon-6 oligomer degradation |
| POLYISOPRENSYN PWY | polyisoprenoid biosynthesis (E. coli) |
| PWY 6113 | superpathway of mycolate biosynthesis |
| PWY 6215 | 4-chlorobenzoate degradation |
| PWY 6285 | superpathway of fatty acids biosynthesis (E. coli) |
| PWY 6292 | superpathway of L-cysteine biosynthesis (mammalian) |
| PWY 6293 | superpathway of L-cysteine biosynthesis (fungi) |
| PWY 6594 | superpathway of Clostridium acetobutylicum solventogenic fermentation |
| PWY 6596 | adenosine nucleotides degradation I |
| PWY 6604 | superpathway of Clostridium acetobutylicum acidogenic and solventogenic fermentation |
| PWY 6612 | superpathway of tetrahydrofolate biosynthesis |
| PWY 7858 | (5Z)-dodecenoate biosynthesis II |
| PWY 801 | homocysteine and cysteine interconversion |
| PWY 822 | fructan biosynthesis |
| PWY 922 | mevalonate pathway I (eukaryotes and bacteria) |

**Supplementary Table 3: GO terms IDs and their description as depicted in Figure 4.**

| **GO** | **Description** |
| --- | --- |
| GO:0000107 | [MF] imidazoleglycerol-phosphate synthase activity |
| GO:0000271 | [BP] polysaccharide biosynthetic process |
| GO:0000725 | [BP] recombinational repair |
| GO:0003916 | [MF] DNA topoisomerase activity |
| GO:0003983 | [MF] UTP:glucose-1-phosphate uridylyltransferase activity |
| GO:0004056 | [MF] argininosuccinate lyase activity |
| GO:0004066 | [MF] asparagine synthase (glutamine-hydrolyzing) activity |
| GO:0004067 | [MF] asparaginase activity |
| GO:0004566 | [MF] beta-glucuronidase activity |
| GO:0006304 | [BP] DNA modification |
| GO:0006464 | [BP] cellular protein modification process |
| GO:0006529 | [BP] asparagine biosynthetic process |
| GO:0008320 | [MF] protein transmembrane transporter activity |
| GO:0008830 | [MF] dTDP-4-dehydrorhamnose 3,5-epimerase activity |
| GO:0008976 | [MF] polyphosphate kinase activity |
| GO:0016020 | [CC] membrane |
| GO:0016051 | [BP] carbohydrate biosynthetic process |
| GO:0016310 | [BP] phosphorylation |
| GO:0016747 | [MF] transferase activity, transferring acyl groups other than amino-acyl groups |
| GO:0016772 | [MF] transferase activity, transferring phosphorus-containing groups |
| GO:0016813 | [MF] hydrolase activity, acting on carbon-nitrogen (but not peptide) bonds, in linear amidines |
| GO:0016830 | [MF] carbon-carbon lyase activity |
| GO:0018662 | [MF] phenol 2-monooxygenase activity |
| GO:0019318 | [BP] hexose metabolic process |
| GO:0030599 | [MF] pectinesterase activity |
| GO:0033281 | [CC] TAT protein transport complex |
| GO:0042545 | [BP] cell wall modification |
| GO:0043039 | [BP] tRNA aminoacylation |
| GO:0043822 | [MF] ribonuclease M5 activity |
| GO:0043953 | [BP] protein transport by the Tat complex |
| GO:0050114 | [MF] myo-inosose-2 dehydratase activity |
| GO:0051912 | [MF] CoB--CoM heterodisulfide reductase activity |
| GO:0102211 | [MF] unsaturated rhamnogalacturonyl hydrolase activity |
| GO:1901135 | [BP] carbohydrate derivative metabolic process |
| GO:0003961 | [MF] O-acetylhomoserine aminocarboxypropyltransferase activity |
| GO:0004149 | [MF] dihydrolipoyllysine-residue succinyltransferase activity |
| GO:0004155 | [MF] 6,7-dihydropteridine reductase activity |
| GO:0004159 | [MF] dihydrouracil dehydrogenase (NAD+) activity |
| GO:0004420 | [MF] hydroxymethylglutaryl-CoA reductase (NADPH) activity |
| GO:0004783 | [MF] sulfite reductase (NADPH) activity |
| GO:0005518 | [MF] collagen binding |
| GO:0006468 | [BP] protein phosphorylation |
| GO:0006486 | [BP] protein glycosylation |
| GO:0006644 | [BP] phospholipid metabolic process |
| GO:0007154 | [BP] cell communication |
| GO:0008417 | [MF] fucosyltransferase activity |
| GO:0008445 | [MF] D-aspartate oxidase activity |
| GO:0009375 | [CC] ferredoxin hydrogenase complex |
| GO:0010038 | [BP] response to metal ion |
| GO:0016799 | [MF] hydrolase activity, hydrolyzing N-glycosyl compounds |
| GO:0016838 | [MF] carbon-oxygen lyase activity, acting on phosphates |
| GO:0016999 | [BP] antibiotic metabolic process |
| GO:0019748 | [BP] secondary metabolic process |
| GO:0033468 | [BP] CMP-keto-3-deoxy-D-manno-octulosonic acid biosynthetic process |
| GO:0033926 | [MF] glycopeptide alpha-N-acetylgalactosaminidase activity |
| GO:0042351 | [BP] 'de novo' GDP-L-fucose biosynthetic process |
| GO:0043211 | [MF] ATPase-coupled carbohydrate transmembrane transporter activity |
| GO:0050462 | [MF] N-acetylneuraminate synthase activity |
| GO:0051787 | [MF] misfolded protein binding |
| GO:0071704 | [BP] organic substance metabolic process |
| GO:1901891 | [BP] regulation of cell septum assembly |
| GO, Gene Ontology; [CC] = Cellular Component; [BP] = Biological Process; [MF] = Molecular Function. | |

**Supplementary Table 4: KEGGs IDs and their description as depicted in Figure 4.**

| **KEGG** | **Description** |
| --- | --- |
| K01190 | beta-galactosidase [EC:3.2.1.23] |
| K01206 | alpha-L-fucosidase [EC:3.2.1.51] |
| K02965 | small subunit ribosomal protein S19 |
| K04751 | nitrogen regulatory protein P-II 1 |
| K00926 | carbamate kinase [EC:2.7.2.2] |
| K00943 | dTMP kinase [EC:2.7.4.9] |
| K00991 | 2-C-methyl-D-erythritol 4-phosphate cytidylyltransferase [EC:2.7.7.60] |
| K01921 | D-alanine-D-alanine ligase [EC:6.3.2.4] |
| K02529 | LacI family transcriptional regulator |
| K02564 | glucosamine-6-phosphate deaminase [EC:3.5.99.6] |
| K02770 | fructose PTS system EIIBC or EIIC component [EC:2.7.1.202] |
| K02959 | small subunit ribosomal protein S16 |
| K03077 | L-ribulose-5-phosphate 4-epimerase [EC:5.1.3.4] |
| K03106 | signal recognition particle subunit SRP54 [EC:3.6.5.4] |
| K03574 | 8-oxo-dGTP diphosphatase [EC:3.6.1.55] |
| K03657 | DNA helicase II / ATP-dependent DNA helicase PcrA [EC:3.6.4.12] |
| K03924 | MoxR-like ATPase [EC:3.6.3.-] |
| K06346 | spoIIIJ-associated protein |
| K06871 | uncharacterized protein |
| K07075 | uncharacterized protein |
| K07107 | acyl-CoA thioester hydrolase [EC:3.1.2.-] |
| K07316 | adenine-specific DNA-methyltransferase [EC:2.1.1.72] |
| K07448 | restriction system protein |
| K07458 | DNA mismatch endonuclease, patch repair protein [EC:3.1.-.-] |
| K10117 | raffinose/stachyose/melibiose transport system substrate-binding protein |
| K11752 | diaminohydroxyphosphoribosylaminopyrimidine deaminase / 5-amino-6-(5-phosphoribosylamino)uracil reductase [EC:3.5.4.26 1.1.1.193] |
| KEGG, Kyoto Encyclopedia of Genes and Genomes; EC = Enzyme Commission number. | |
